# New strategies of neurodegenerative disease treatment with extracellular vesicles (EVs) derived from mesenchymal stem cells (MSCs)

**DOI:** 10.7150/thno.83066

**Published:** 2023-07-16

**Authors:** Chella Perumal Palanisamy, JinJin Pei, Phaniendra Alugoju, Naga Venkata Anusha Anthikapalli, Selvaraj Jayaraman, Vishnu Priya Veeraraghavan, Sridevi Gopathy, Jeane Rebecca Roy, Coimbatore Sadagopan Janaki, Dwarakesh Thalamati, Monica Mironescu, Qiang Luo, Yu Miao, Yuan Chai, Qianfa Long

**Affiliations:** 1 Mini-invasive Neurosurgery and Translational Medical Center, Xi'an Central Hospital, Xi'an Jiaotong University, No. 161, West 5th Road, Xincheng District, Xi'an, 710003, PR China.; 2 Centre of Molecular Medicine and Diagnostics (COMManD), Department of Biochemistry, Saveetha Dental College & Hospital, Saveetha Institute of Medical & Technical Sciences, Saveetha University, Chennai 600077, India.; 3 Qinba State Key Laboratory of Biological Resources and Ecological Environment, 2011 QinLing-Bashan Mountains Bioresources Comprehensive Development C. I. C, Shaanxi Province Key Laboratory of Bio-Resources, College of Bioscience and Bioengineering, Shaanxi University of Technology, Hanzhong 723001, China.; 4 Department of Clinical Chemistry, Chulalongkorn University, Bangkok 10330, Thailand.; 5 Department of Chemistry, A.N.R College, Bethavolu, Gudivada, Andhra Pradesh 521301, India.; 6 Department of Physiology, SRM Dental College, Ramapuram campus, Chennai, Tamil Nadu 600089, India.; 7 Department of Anatomy, Bhaarath Medical College and hospital, Bharath Institute of Higher Education and Research (BIHER), Chennai, Tamil Nadu 600073, India.; 8 Department of Anaesthesia, Hull Royal Infirmary, NHS, England.; 9 Faculty of Agricultural Sciences Food Industry and Environmental Protection, Lucian Blaga University of Sibiu, Bv. Victoriei 10, 550024 Sibiu, Romania.

**Keywords:** MSC-EVs, Neurodegenerative diseases, Mechanisms, Therapeutic strategies

## Abstract

Neurodegenerative diseases are characterized by the progressive loss of neurons and intricate interactions between different cell types within the affected regions. Reliable biomarkers that can accurately reflect disease activity, diagnose, and monitor the progression of neurodegenerative diseases are crucial for the development of effective therapies. However, identifying suitable biomarkers has been challenging due to the heterogeneous nature of these diseases, affecting specific subsets of neurons in different brain regions. One promising approach for promoting brain regeneration and recovery involves the transplantation of mesenchymal stem cells (MSCs). MSCs have demonstrated the ability to modulate the immune system, promote neurite outgrowth, stimulate angiogenesis, and repair damaged tissues, partially through the release of their extracellular vesicles (EVs). MSC-derived EVs retain some of the therapeutic characteristics of their parent MSCs, including their ability to regulate neurite outgrowth, promote angiogenesis, and facilitate tissue repair. This review aims to explore the potential of MSC-derived EVs as an emerging therapeutic strategy for neurodegenerative diseases, highlighting their role in modulating disease progression and promoting neuronal recovery. By elucidating the mechanisms by which MSC-derived EVs exert their therapeutic effects, we can advance our understanding and leverage their potential for the development of novel treatment approaches in the field of neurodegenerative diseases.

## 1. Introduction

There is an increasing prevalence of neurodegenerative diseases among an aging population, which motivates biomedical scientists to investigate these conditions, such as Alzheimer's disease, Parkinson's disease, multiple sclerosis (MS), amyotrophic lateral sclerosis (ALS), and Huntington's disease (HD) 1. Chronic neurodegenerative processes can also be exacerbated by acute injuries and can increase the levels of systemic neuroinflammatory responses by affecting the central nervous system (CNS) 2. According to the World Health Organization (WHO), neurological disorders are expected to become the second leading cause of death in humans within the next 20 years 3. To overcome the failure of current therapeutic options, new therapeutic strategies must be developed to combat neurodegenerative diseases 4. There is a lack of suitable carriers for targeted drugs, which prevents many advanced compounds from being fully developed 5.

A neurodegenerative disease leads to a gradual loss of neurons and a decline in their functionality, resulting in cognitive and behavioural impairments 6. Glial cells or neuroglia are the non-neuronal cells of the central nervous system. Neuroglia are categorized into four types such as astrocytes, microglia, oligodendrocytes, and NG2a-glia, all of these are involved in the regulation of the brain plasticity, protection of neurons, homeostasis 7. Neurons, microglia, astrocytes, and oligodendrocytes interact with one another in a way that further damages the central nervous system, resulting in neuron and myelin death 8.

In recent years, inflammation-related activated astrocytes have gained increasing attention in relation to neurodegenerative diseases 9-11. In several studies, it has been shown that the presence of type 1 astrocytes (neurotoxic phenotype) that secrete proinflammatory cytokines has been associated with neurodegenerative diseases 12-14. Furthermore, an increased number of activated microglia cooperated with dysfunctional astrocytes to impair the survival of neurons in AD mouse models 15. Dysfunctional microglia play a very important role in neurodegeneration, and this is well known 16. In AD and ALS, single cell sequencing analysis revealed that there was an increase in the expression level of several genes relevant to microglia function as the disease progressed 17.

A central nervous system oligodendrocyte produces axon's myelin sheath. Axons are protected by the myelin sheath, which maintains the axon's environment and function 18. During demyelination, the insulation of nerve fibres is lost, causing the conduction of nerve signals to be disrupted, and the survival of the axons to be compromised 19. The typical demyelinating diseases include multiple sclerosis and neuromyelitis optica, which are both related to demyelination 20. The oligodendrocyte precursor cells (OPCs) that generate new myelin sheaths require proliferation, migration into lesions, and differentiation into oligodendrocytes. When these diseases progress, however, remyelination is often unsuccessful 21. There is a pressing need to develop a new method that promotes myelin regeneration.

Neurodegenerative diseases encompass a range of conditions where neurons in the brain or peripheral nervous system progressively degenerate. Although most neurodegenerative diseases like Alzheimer's, Parkinson's, and Huntington's lack a cure, there are limited treatment options available 22. Current methods include medications, physical and occupational therapy, speech therapy, assistive devices, and lifestyle adjustments, which can aid in symptom management and slow disease progression. It's crucial to understand that while these treatments improve symptoms and enhance quality of life, they do not halt or reverse the disease's advancement. Ongoing research is dedicated to developing therapies that can modify the underlying degenerative processes and decelerate the progression of these conditions 23.

Physiologically relevant information is transported into extracellular spaces by membrane-bound vesicles, which are formed by cells or directly from their membranes 24. EVs are primarily designed to facilitate communication between cells without requiring direct contact 25-27. They perform a variety of physiological and pathological functions beyond those released during apoptosis. In addition to their therapeutic potential, extracellular vesicles (EVs) derived from mesenchymal stem cells (MSCs) can also carry products that are specifically related to the pathological stage of neurological diseases. This characteristic makes them attractive as potential diagnostic tools and targets for personalized medicine approaches. 28. Genotyping and phenotyping can be altered by the transfer of vesicular proteins, lipids, and genetic materials (such as DNA, RNA, and miRNA) 29. EVs interact with target cells by recognizing and interacting with their receptor on the surface. EVs are released by a variety of neuronal subtypes, such as microglia, astrocytes, and Schwann cells 30. Drugs can be administered more efficiently with EVs since they cross the blood-brain barrier directly, maximizing their efficacy. EVs are being tested as potential treatment methods for neurodegenerative diseases 31.

## 2. Neurodegenerative diseases and their characteristics

**Figure 1:**
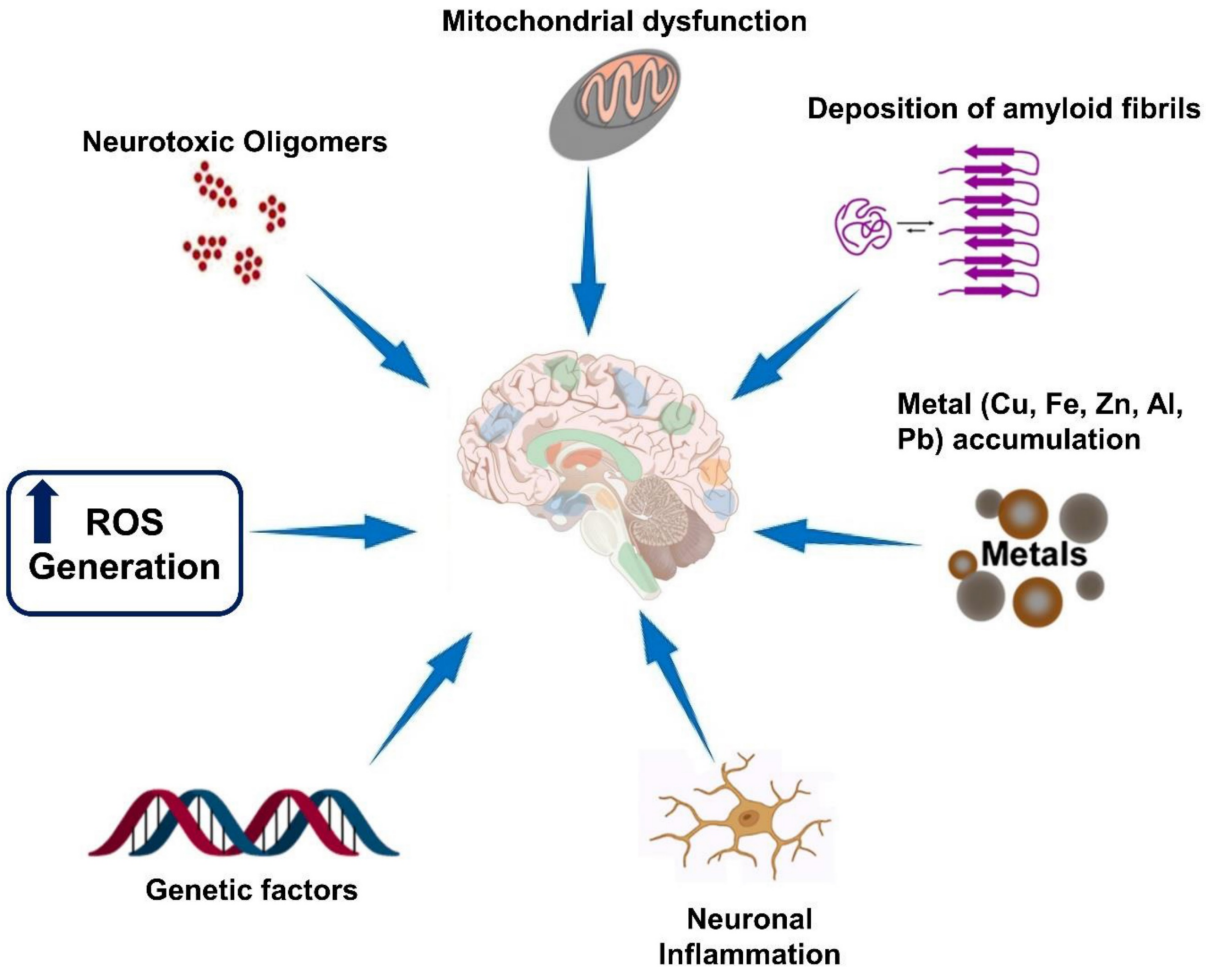
** Hallmarks of neurodegenerative diseases.** Neurodegenerative diseases are a group of disorders characterized by the progressive degeneration and dysfunction of neurons in the central nervous system. While each neurodegenerative disease has its own unique features, there are several common hallmarks that are often associated with these conditions.

Neurodegenerative diseases are a group of disorders characterized by the progressive loss of structure and function of neurons in the brain and, in some cases, the peripheral nervous system. While each neurodegenerative disease has its own unique features, there are several hallmarks that are commonly observed across these disorders which are illustrated in Figure 1. It is important to note that the specific hallmarks and disease mechanisms can vary among different neurodegenerative diseases, and ongoing research aims to uncover further insights into these complex disorders 32.

BBB prevents peripheral immune cells from passing through the brain, making it an “immunologically privileged” organ 33. Several glial cells, including microglia and astrocytes, interact with the immune system within the peripheral nervous system 34. When activated, microglia release ROS, NO, and inflammatory cytokines, including IL-1β, IL-6, and tumor necrosis factor (TNF), leading to neuroinflammation 35. Activated microglia, proinflammatory mediators, and increased oxidative and nitrosative stress all contribute to chronic inflammation and neurodegenerative diseases 36. Microglia, the resident immune cells of the central nervous system, play a complex role in maintaining brain homeostasis, responding to injury, and regulating the immune response in the brain. While microglia can contribute to the death of excitatory neurons in certain conditions, it is essential to note that microglia's role in neuronal death is context-dependent and can vary across different diseases and stages of neurodegeneration. Microglia are also responsible for the death of excitatory neurons resulting in the development of neurodegenerative diseases such as AD and ALS 37. Numerous signaling molecules are released by degenerating neurons along with nucleotides, cytokines, and chemokines 38. On the other hand, neuroinflammatory pathways are also mediated by astrocytes 39. Inflammatory stimuli, however, seem to be largely passive for microglia. The activation of microglia results in the transformation of astrocytes into harmful A1 cells. High levels of A1 astrocytes are reported in several neurodegenerative diseases, such as AD, PD, ALS, and multiple sclerosis, A1 astrocyte levels are high 40.

Besides the infiltration of inflammatory cells into the CNS, demyelination is another important hallmark of neurodegenerative diseases. Demyelination of axons occur due the death of oligodendroglia, infectious agents, overreactions of the immune system, and trauma 41. Thus, the loss of myelin sheath ultimately affects the distribution of ion channels including voltage gated sodium channels (Nav channels) subsequent development of axonal transport disorders 42. At the distal end of an injured axon, oligodendrocytes degenerate due to lack of nutrition. Damaged oligodendrocytes are replaced by new oligodendrocytes, and spared axons are remyelinated by neural stem cells (NSCs) 43. The spontaneous remyelination of oligodendroglial precursor cells (OPCs) and NSCs after axonal injury is affected by several factors, including the downregulation of trophic growth factors 44. Thus, the failure to regenerate myelin is primarily a result of: Lack of key growth factors involved in oligodendrocyte maturation and myelin formation; Inadequate removal of myelin fragments 45. When inflammatory cells infiltrate and activate at the site of the lesion, further demyelination, axonal mutation, and neuronal dysfunction occur 46. Nkx2.2, Olig2, and Sox2 are upregulated by demyelination injury to stimulate the differentiation of OPCs 47. Growth factors are also released from the lesion area, including platelet-derived growth factor (PDGF) and fibroblast growth factor (FGF) 48. The differentiation of oligodendrocytes produces myelin proteins, including Myelin basic protein (MBP), that increase the density of the myelin membrane 49. Overactive macrophages and microglia may lead to immunotoxicity, although they play an important role in the process. Microglia and macrophages may remove myelin fragments from the lesion area, inhibiting OPC growth 50. MBP is a myelin protein found in macrophages and microglia. As a result, it is imperative to note in short, inflammatory cells can infiltrate and demyelinate in neurodegenerative diseases 51. Axons damaged by axonal injury do not have the proper structure and physiological characteristics to be repaired by remyelination achieved by CNS precursor cells 52.

### 2.1. Traditional therapies for neurodegenerative disease

Traditional therapies for neurodegenerative diseases primarily focus on managing symptoms and slowing down disease progression. Here are some commonly used traditional therapies for neurodegenerative diseases:

**Pharmacological Treatments:** Medications are often prescribed to manage symptoms associated with neurodegenerative diseases. For example, levodopa is commonly used in Parkinson's disease to replenish dopamine levels and alleviate motor symptoms. Cholinesterase inhibitors, such as donepezil, are used in Alzheimer's disease to enhance cognitive function. There are also medications to manage symptoms like muscle stiffness, tremors, and sleep disturbances 53.

**Physical and Occupational Therapy:** Physical and occupational therapy are beneficial for improving mobility, strength, balance, and coordination in neurodegenerative diseases. These therapies may involve exercises, stretching, gait training, and assistive devices to help maintain functional independence and quality of life 54.

**Speech and Swallowing Therapy:** Neurodegenerative diseases can affect speech and swallowing functions. Speech therapy can help individuals with speech difficulties by teaching techniques to improve articulation and strengthen vocal muscles. Swallowing therapy, on the other hand, focuses on strategies to prevent aspiration and maintain safe and efficient swallowing 55.

**Supportive Care:** Supportive care involves addressing the non-medical needs of individuals with neurodegenerative diseases. It includes assistance with daily activities, emotional support, counseling, and education for both patients and their caregivers. Support groups and community resources can also provide a network of support and information 56.

**Assistive Devices:** Various assistive devices can enhance independence and quality of life for individuals with neurodegenerative diseases. Examples include mobility aids like canes, walkers, and wheelchairs, as well as devices that assist with communication, such as speech-generating devices or eye-tracking technology 57.

**Lifestyle Modifications:** Adopting a healthy lifestyle can contribute to overall well-being in neurodegenerative diseases. This may include regular exercise, a balanced diet, stress management techniques, and sufficient sleep. Maintaining social connections and engaging in mentally stimulating activities can also be beneficial.

It's important to note that while traditional therapies can help manage symptoms and improve quality of life, they do not offer a cure for neurodegenerative diseases. Research is ongoing to develop disease-modifying therapies and explore innovative approaches, such as stem cell therapies and gene therapies, to address the underlying causes of these diseases 58.

### 2.2. Defects in traditional treatments for neurodegenerative diseases

Traditional therapies for neurodegenerative diseases have limitations and drawbacks. These include their limited efficacy in slowing disease progression and providing long-term benefits, the significant side effects associated with pharmacological treatments, their inability to modify the course of the disease, the non-personalized approach that overlooks individual variations, the incomplete management of symptoms, and the lack of curative potential. To overcome these limitations, ongoing research focuses on the development of novel therapies such as stem cell-based treatments, gene therapies, immunotherapies, and targeted therapies. These advancements aim to provide more effective and disease-modifying approaches for neurodegenerative diseases 59-62

### 2.3. Some advanced therapies for neurodegenerative diseases

**Figure 2:**
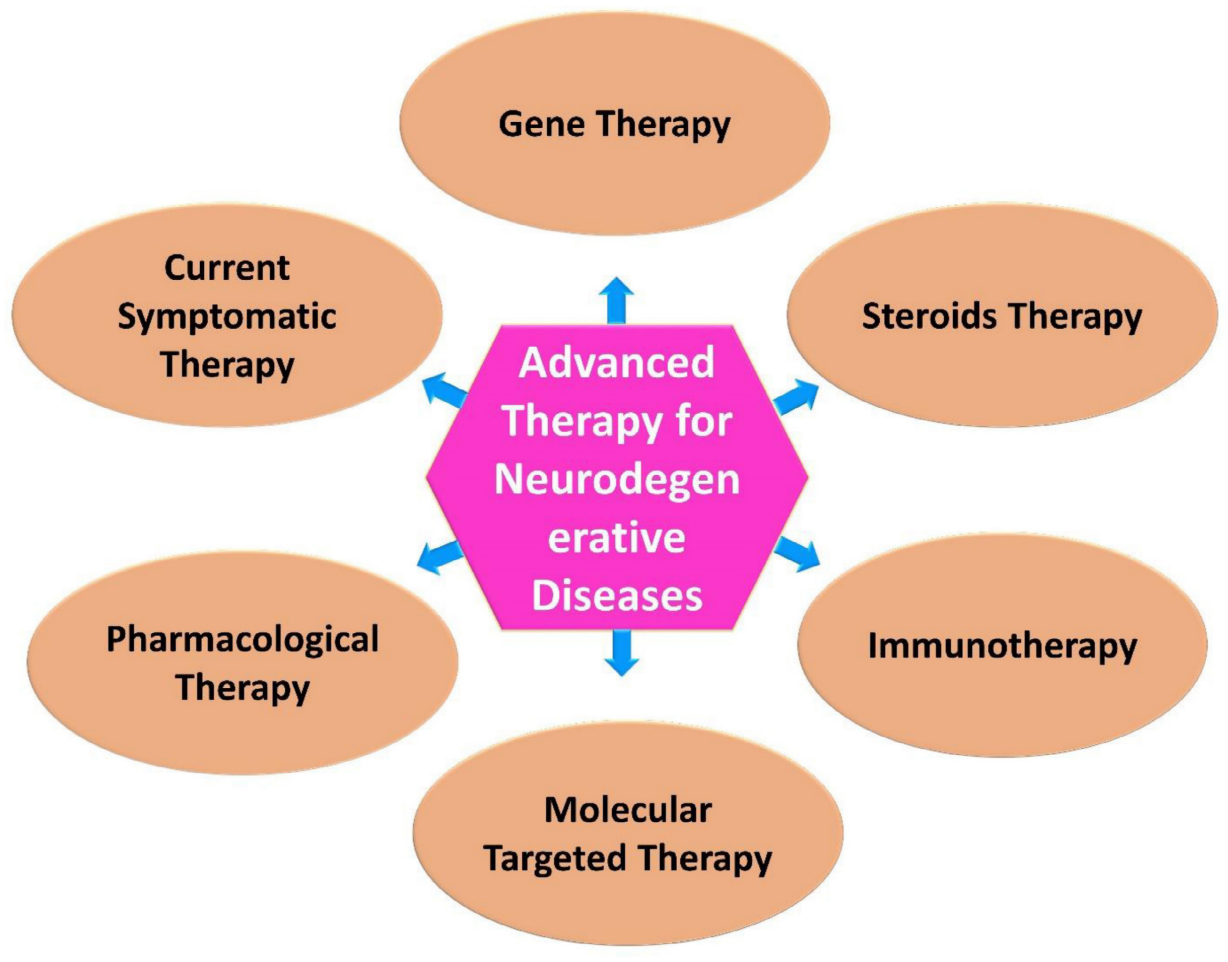
** Currently available therapies for neurodegenerative diseases.** While there is no definitive cure for most neurodegenerative diseases, there are various therapies and interventions available to help manage symptoms, slow disease progression, and improve the quality of life for individuals affected by these conditions.

Neurological research is expected to benefit from advances in biomedical research, especially in the development of newer and more targeted therapeutic approaches. In medicine and therapeutics, modern approaches to treating diseases have become increasingly popular in recent years 63. Advanced technologies are developing, being applied in therapy, undergoing regulatory review, and being monitored post-approval, and a variety of concerns are emerging with regard to their development, application and regulation (Figure 2) 64.

#### 2.3.1. Gene therapy

Gene therapy may be one of the several beneficial ways to treat neurodegenerative diseases 65. Through understanding of the underlying diseases mechanism through the regulation of gene expression at spatial and temporal level is crucial to develop effective therapeutic interventions against neurodegenerative diseases 66. However, preventing leakage into neighboring regions or perivascular spaces is one of the challenging aspects of gene therapy by transduction. Gene therapy involves delivering therapeutic genes to target cells or tissues to correct genetic disorders, provide therapeutic effects, or modulate cellular functions. Transduction refers to the process of introducing the therapeutic genes into the target cells 67. Real-time monitoring of vector delivery has become the gold standard for gene therapy through MRI-guided convection-enhanced delivery (iMRI-CED) 68. It would be possible to translate promising preclinical therapies for neurodegenerative disorders into clinical trials if this advanced neurosurgical technique is successfully applied 69.

#### 2.3.2. Steroids

Amelioration of neuronal function may constitute the one of the preventive strategies to fight against neurodegenerative diseases 70. The improvement of neuronal function can be achieved by the application of neuroactive steroids and sex steroids which promote survival, neurogenesis, and memory function by limiting neuronal apoptosis, oxidative stress, mitochondrial dysfunction, and microglial activation 71. The beneficial effects of these steroids depend on the sex and stage of neuropathology of a patient 72. Besides, the current research on the use of steroids for the treatment of neurodegenerative diseases is still limited 73. In fact, several factors need to be considered before the use of steroids for the treatment of neurodegenerative diseases include formulation, dosage, rout of administration, bioavailability, etc. 74.

#### 2.3.3. Immunotherapies

Immunotherapy by the application of monoclonal antibody and specific antigen may play a role in managing the progressive neurodegenerative diseases as most of them associated with accumulation of misfolded and aggregated proteins 75. Growing evidence suggest that harnessing the immune system might be a promising solution. Neurodegenerative diseases associated with aging and non-autoimmune conditions represent a significant healthcare challenge, yet they have received relatively limited attention in the context of immunotherapies. While immunotherapies have shown promise in autoimmune disorders, their potential in these specific neurodegenerative diseases remains largely untapped. The complex nature of these diseases, involving multiple pathological mechanisms and intricate interactions between aging, neuroinflammation, and neuronal dysfunction, presents unique challenges for immunotherapy development. 76. Recent advances in immunomodulation in the central nervous system have enabled us to gain a deeper understanding of how they work. Exploring novel targets for immunotherapy strategies is an important avenue of research in the field of medicine. While conventional disease biomarkers have been valuable for diagnostic and therapeutic purposes, identifying and targeting new immune-related targets can offer additional opportunities for developing innovative immunotherapies. 77. The potential for more effective treatment appears to lie in immunotherapy targeting abnormal protein aggregates or inflammatory molecules 78.

#### 2.3.4. Molecular targeted therapies

Molecular targeted therapy for neurodegenerative diseases has been developed in advanced neurobiology 79. It has been shown that several compounds are effective in animal studies, but they have not been proven to be effective in human trials. In both basic and clinical research, it is important to examine the efficacy of potential agents that might modify disease progression 80. To improve the credibility of preclinical studies, it is crucial that positive results from animal experiments be replicated 81. A clinical outcome measure must be developed that withstands variability, subjectivity, and placebo effects due to the modest efficacy of molecular targeted therapies in humans 82. A growing number of interventions are also being tested prior to symptom onset. This type of preventive trial can benefit from natural histories of biological and neurophysiological markers. Conceptual innovation is necessary for both basic and clinical research since conventional approaches do not always work for molecular targeted therapies 83.

#### 2.3.5. Pharmacological Therapy

The anti-beta-amyloid, nerve growth factor, and anti-inflammatory properties of drugs under study or awaiting approval for AD treatment are of particular interest. By modulating secretase activity and enhancing beta-amyloid precursor protein synthesis, some drugs inhibit beta-amyloid production in addition to inhibiting acetylcholinesterase 84. In addition to immunotherapy, metal ions' interactions with beta-amyloid and oxidative reactions can be controlled, as well as metabolic and hormonal control. It is expected that several dopamine uptake inhibitors and glutamate AMPA receptor antagonists will be registered for the treatment of PD symptoms 85. The class of drugs that inhibit dopamine uptake includes antagonists of the adenosine A(2A) receptor, inhibitors of monoamine oxidase B, and ion channel modulators. In addition, alpha(2)-adrenergic receptor antagonists, 5-HT(1A) agonists, and astrocyte-modulating agents are also used for treating PD-related dyskinesias or dyskinesias associated with l-DOPA 86. By stabilizing the membrane and maintaining mitochondria, LAX-101 demonstrates antiapoptotic properties as part of Huntington's disease treatment. Drugs with antioxidant, antiapoptotic, and neuroprotective properties are being used to treat neurodegenerative diseases with less common symptoms 87.

#### 2.3.6. Current symptomatic therapies

The FDA has approved four cholinesterase inhibitors (ChE-Is) and one N-methyl-D-aspartate (NMDA) receptor antagonist for treating AD 88. The three ChE-Is that are currently available are donepezil, rivastigmine, and galantamine. The NMDA receptor is antagonistic by memantine. These drugs enhance cognitive performance. Memantine and cholinesterase inhibitors have similar effects on cognition, global function, and activities of daily living 89. In some cases, neuropsychiatric symptoms can be ameliorated and new symptoms can be prevented through the administration of symptomatic agents 90. Unlike other neurodegenerative diseases, AD results in the loss of presynaptic cholinergic neurons in Meynert's nucleus basalis, while post-synaptic cholinergic neurons remain. In conjunction with cognitive improvements, cholinergic augmentation can be used to achieve functional post-synaptic stimulation 91. Amygdala and cerebral cortex receive their acetylcholine primarily from the nucleus basalis. Cortical projections to the nucleus basalis are strongly correlated with cholinergic input to limbic and paralimbic cortices 92. In other transmitter systems crucial to cognition, pre-/post-synaptic disconnection has not been identified, which explains why other transmitter systems do not enhance cognition. Intact circuits are essential to cognitive function, which includes both cholinergic innervation and the involvement of other transmitters. During ChE-Is treatment, cortical circuit activity is usually increased in fMRI or fluorodeoxyglucose positron emission tomography (PET) 93. In developing more effective treatments, new imaging tools may assist in improving cognitive function 94.

## 3. Therapies of MSCs in neurodegenerative disease

Mesenchymal stem cell (MSC) therapies have shown promise in the field of neurodegenerative diseases. MSCs are multipotent stem cells that can differentiate into various cell types, including neurons and glial cells. They also possess immunomodulatory and trophic properties that can aid in tissue repair and regeneration. Here are some of the therapies involving MSCs that have been explored for neurodegenerative diseases:

**Parkinson's Disease (PD):** MSCs have been investigated as a potential treatment for PD. They can be transplanted into the brain to replace lost dopaminergic neurons or release neurotrophic factors that promote the survival of existing neurons. Clinical trials have demonstrated the safety and potential efficacy of MSC transplantation in PD patients 95.

**Alzheimer's Disease (AD):** MSCs have been studied as a therapeutic option for AD. They can secrete neurotrophic factors and anti-inflammatory molecules, which may help reduce neuroinflammation and promote neuronal survival. MSC-based therapies have shown positive effects in animal models of AD, but clinical trials are still in the early stages.

**Amyotrophic Lateral Sclerosis (ALS):** MSCs have been investigated as a potential treatment for ALS, a progressive neurodegenerative disease affecting motor neurons. MSCs can be delivered either through direct injection into the spinal cord or intravenously. They have shown beneficial effects in preclinical studies, including improved motor function and prolonged survival in animal models of ALS.

**Huntington's Disease (HD):** MSCs have been explored as a potential therapy for HD, an inherited neurodegenerative disorder. Studies have shown that MSCs can secrete growth factors and promote neuroprotection in animal models of HD. However, further research is needed to determine the safety and efficacy of MSC-based therapies in humans.

**Multiple Sclerosis (MS):** MSCs have been investigated as a treatment for MS, an autoimmune disease characterized by inflammation and demyelination of the central nervous system. MSCs can modulate the immune response and promote tissue repair. Clinical trials have shown that MSC transplantation can improve symptoms and reduce disease activity in MS patients.

It is important to note that while MSC-based therapies hold promise, more research is needed to fully understand their mechanisms of action, optimize delivery methods, and ensure their long-term safety and efficacy in treating neurodegenerative diseases. Clinical trials are ongoing, and regulatory approval is required before these therapies become widely available 96.

## 4. Clinical applications and characteristics of mesenchymal stem cells (MSCs)

Generally, stem cells are non-committed, non-educated, and uncommitted cells. In addition to self-renewing, similar, and differentiating cells, these cells can also produce different types of cells. A variety of tissues can be restored using stem cells due to their unique properties. There are three types of stem cells: embryonic stem cells (ESCs), induced pluripotent stem cells (iPSCs), and adult stem cells. Friedenstein described MSCs for the first time in 1974 97. MSCs are considered adult stem cells from the viewpoint of stem cells. Research has shown that, MSCs possess several characteristics that make them unique among stem cells, including the ability to self-renew, the ability to differentiate into a multipotent state, the ability to act as immunomodulators, and the ability to act as non-immunogenic cells. It has been described that a stem cell structure resembles a fibroblast, according to the International Society for Stem Cell Research (ISSCR) 98. In culture, a variety of tissues can be expanded, including bone marrow, muscle, adipose tissue, chorionic villi, menstrual blood, and dental pulp 99. In an *in vitro* culture system, MSCs can continue to grow and give rise to multiple lines of cells while retaining their ability to divide and give rise to multiple lines of cells. There are several surface markers that can be used to characterize MSCs 100.

The immunogenicity of MSCs is relatively low, and they also possess immunomodulatory properties 101. Consequently, MSCs are a promising therapeutic and regenerative medicine candidate for a variety of diseases 102. In addition, MSCs are able to restore tissues after injury, aid in tissue regeneration, and maintain the equilibrium of tissues. The MSCs have also been shown to be capable of altering the mechanisms of innate and adaptive immunity by interfering with the proliferation and activation of immune cells 103. A significant number of MSCs did not proliferate following MSC transplantation in clinical trials, raising the question of whether MSCs are effective 104. Cell injury is reduced and tissue repair capacity is improved as a result of the collegial action of molecules secreted by MSCs. As soluble molecules are transported to their targets, it is not necessary for parent cells to be contiguous. The vast potential of MSCs is now being explored, despite their use during the prephases for local engraftment and differentiation into different types of tissue. A variety of biologically active factors are produced by these mediators in all biological processes 105.

The current understanding of MSC-based therapies has been supplanted by a new type of biological directive that makes use of the secreted materials of the cells 106. In order for cells to function properly, an individual's environment must play a crucial role. Secretomes of cells contain a number of molecules that support cell proliferation and ensure efficient performance of vital functions 107. As part of their normal physiological functions, secretory molecules aid in cell-to-cell interactions in growth culture medium. Secretomes are composed of proteins, enzymes, organic compounds, growth factors, cytokines, metabolites, neurotransmitters, and hormones. There is a general belief that these substances secreted affect the extracellular matrix and its structure and content. Any cell can be examined using these components to determine its underlying conditions 108. Compared to direct cell therapies, conditioned media and exosomes have substantial advantages due to their low maintenance and longer shelf lives. Absolutely, the manufacture and quality control of EVs must also meet certain statutory requirements to ensure their safety and efficacy as therapeutic products. Just as with cell-based products, the development, production, and evaluation of EV-based therapies involve stringent regulatory considerations 109.

MSCs produce a number of transcription factors (TFs) that encourage neural cell growth and survival (for example, in cases of spinal cord injuries) 110. Neurological disorders can be treated with MSCs via their soluble TFs contained within their secretome. As a result of the paracrine hypothesis, the secretome is believed to possess regenerative potential. According to this hypothesis, stem cells release substances that help heal damaged or diseased tissue 111. The secretome of MSCs holds promise as a potential alternative to invasive surgical procedures for treating various conditions. The secretome refers to the complex mixture of bioactive molecules, including growth factors, cytokines, chemokines, extracellular vesicles, and other signaling molecules, released by MSCs. While the use of the MSC secretome holds promise, it's important to note that further research and clinical studies are needed to fully understand its mechanisms of action, optimize delivery methods, determine appropriate dosing, and evaluate its safety and efficacy across different conditions. Nonetheless, the MSC secretome represents an exciting avenue for non-invasive therapeutic approaches, potentially offering alternatives to conventional invasive surgical procedures in certain clinical scenarios 112. It has been demonstrated in several studies that the MSC secretome facilitates tissue repair through the prevention of apoptosis, the regulation of inflammatory responses, and the enhancement of housekeeping repair mechanisms such as neurogenesis 105.

## 5. Extracellular Vesicles (EVs)

**Figure 3:**
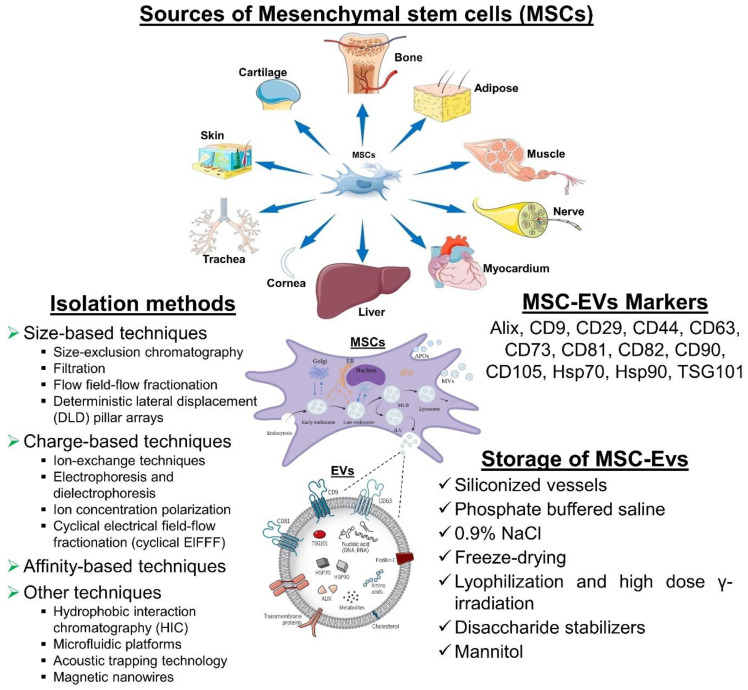
** MSC-derived EVs for neurodegenerative diseases.** MSC-derived EVs have gained significant attention as potential therapeutic agents for neurological diseases. It represents some information on their sources, isolation methods, biomarkers, and storage considerations. However, current researches are still evolving, and there may be additional developments and refinements in isolation methods, biomarker identification, and storage strategies as further research progresses.

The EV is composed of heterogeneous nanoscale vesicles released by a variety of cell types (Figure 3) 113. They are classified as subtypes into exosomes, microvesicles, and apoptotic bodies and can transport protein, mRNA, miRNA, DNA, and lipids within the cell 114. The size and biogenic pathways of these subtypes can help distinguish them. A typical exosome is below 100 nm in diameter and is one of the smallest vesicles 115. Exosomes are released after the plasma membrane fuses with multivesicular endosomes (MVEs), as well as from intraluminal budding within endosomes 116. Extracellularly soluble proteins as well as cell surface proteins form a goblet structure upon first invasion. When endosomes are sorted early in the process, early sorting endosomes (ESEs) are formed, which can sometimes merge with pre-existing ESEs 117. ESEs are also formed and enriched in trans-Golgi networks and endoplasmic reticulum. A multi-vesicular body (MVB) is produced when ESEs become late-sorted endosomes (LSEs) 118. When MVBs fuse with lysosomes or autophagosomes, they are degraded, or through fusion with plasma membranes, they are released as exosomes 119. Microvesicles, on the other hand, range in size from 50 nanometers to one micrometer which are derived from plasma membranes. Apoptotic bodies are heterogeneous vesicles released during apoptosis 120. The lack of subtype-specific markers and overlaps in vesicle sizes have resulted in the absence of a standard nomenclature for EVs 121. Thus, different types of vesicles are difficult to distinguish.

EVs can be released in response to stress, reactive oxygen species, or hypoxia. Since exosomes are derived from MVBs, they contain late endosome proteins, such as CD63, LAMP1, and LAMP2 122. A variety of mechanisms may affect the exocytosis of vesicles, including the early endosome proteins Rab4 and Rab5 123. There are a variety of mechanisms involved with EV uptake, including protein-protein interactions, endocytosis, membrane fusion, and cell-specific mechanisms. Protein-protein interactions between EV proteins and membrane receptors on target cells have been demonstrated. As well as adhesion, motility, activation, and proliferation of cells, tetraspanins play a number of other functions 124. On the surface of EVs, they are abundant, suggesting they contribute to their adoption. In addition to its role as a tetraspanin, Tspan8 is known to interact with integrins 125. Aortic endothelial cells absorb EVs containing Tspan8-CD49d complexes induced by overexpression of Tspan8 126. Endosomes are also generally involved in the endocytosis of EVs. In only 15 minutes after EVs are introduced into the cell, they can be detected intracellularly 122, 127.

A requirement for the uptake of EVs is clathrin-mediated endocytosis (CME). Chlorpromazine inhibits CME, which results in a decreased ability of recipient cells to absorb EVs. The uptake of macrophages is also accompanied by EV colocalization with clathrin 128. Eukaryotic cells are capable of clathrin-independent endocytosis through caveolin-dependent endocytosis (CDE) 129. Phagocytosis is another method of internalizing EVs. Phosphocytic activity is influenced by PI3Ks, which are known to play a key role. There is a dose-dependent inhibition of PI3K by LY294002 and wortmannin during EV uptake 130. As a third method, EVs are directly fused with the membranes of cells. The fusion of EVs with plasma membranes or endosomes can also be detected by delivering miRNAs and luciferin to recipient cells 131. In exocytosis, endocytosis, and cell-cell adhesion, AnxA2 is an essential phospholipid-binding protein 132. It has been shown that EVs containing AnxA2 are capable of fusing directly with plasma membranes 116, 133.

The uptake of EVs by cells is another method of entry. Endothelial cells and pancreatic cells internalize a greater proportion of lymph node stroma-derived EVs in Tspan8, while parental lymph node stroma cells internalize a lesser percentage 134. Furthermore, peritoneal exudate cells are more efficient at internalizing EVs derived from pancreatic adenocarcinoma than granulocytes or T cells. Cells may perform several functions after EVs have been absorbed. Communication between cells is facilitated by EVs. Incorporating into the plasma membrane can be achieved via direct fusion with the plasma membrane or by endocytosis 135. EVs can also transport biomolecules over long distances, making them effective biological carriers. There are several physiological and pathological processes associated with this process. As biological carriers, EVs offer a number of advantages. EVs offer unique opportunities for both cell production and the delivery of endogenous drugs. Cells can be delivered drugs or oligonucleotides by repackaging them into secretory vesicles using this method 136. Drug-loaded vesicles can be produced, loaded, and released by cells, simplifying and improving the loading process. EVs may also be able to serve as biomarkers for different diseases due to the changing expression of specific proteins within circulating EVs 137. The transmission of information is another function of EVs in the central nervous system. EVs are responsible for regulating synaptic activity, forming myelin sheaths, and repairing damaged neurons 138. Due to engineering modifications, EVs are now more convenient and perform better for clinical applications such as, enhanced cargo loading efficiency, Targeting capabilities, Stability and protection of cargo, Scalable production methods and Safety and immunogenicity considerations. It's important to note that while engineering modifications have improved the convenience and performance of EVs for clinical applications, challenges still exist, including standardization of manufacturing processes, optimization of cargo loading, and establishing robust quality control measures. It is possible to identify and track EVs throughout the body using fluorescent imaging, bioluminescence imaging, nuclear imaging, and tomography. In particular, EVs are highly promising candidates for treating CNS disorders since they have the ability to cross the BBB 139.

### 5.1. Isolation of MSC-EVs

It is not possible to isolate EVs in a standardized or unique way. There might be a reason for the variability in characteristics and bioactivities between laboratories when it comes to EVs. Isolating EVs with high yield and purity while preserving their structure and activity is the challenge for clinical applications. Moreover, the isolation method should demonstrate scalability, cost-effectiveness, compatibility with high-throughput production, and ideally, be enclosed 140.

#### 5.1.1. Size-based techniques

##### 5.1.1.1. Size-exclusion chromatography

A well-established method for the separation of macromolecules is size exclusion chromatography (SEC), which is based on the size of the molecules or their hydrodynamic volumes. In a typical SEC system, a porous stationary phase is used for chromatographic separation, coupled either to a pump for elution or to the stationary phase for chromatographic separation. Cell culture-derived samples, blood-derived samples, and other samples containing EVs have been isolated using SEC using a large variety of sample matrices from both prokaryotes and eukaryotes 141.

##### 5.1.1.2. Filtration

For the purification of EVs, filtration is another popular size-based separation method. Most studies use ultrafiltration membranes with molecular weight cut-offs ranging from 10 to 100 kDa. A wide range of samples, including urine and cell cultures, can be isolated from relatively dilute samples using ultrafiltration (UF). In general, UF devices are comprised of a membrane inserted within a container. Centrifugal UF is often used for filtration, which offers the advantage of being relatively simple and easy to use, as well as providing faster EV isolation than UC methods 142.

##### 5.1.1.3. Flow field-flow fractionation

There is an emerging size-based fractionation technique for EV separation known as flow field-flow fractionation (FFF), with asymmetrical flow field-flow fractionation (AsFlFFF or AF4) being the most commonly used FFF subtechnology. The diffusion coefficients of macromolecules are used to separate them in AsFlFFF 143.

##### 5.1.1.4. Deterministic lateral displacement (DLD) pillar arrays

The DLD technique involves the separation of EVs according to their trajectory in a pillar array. The size of the particles smaller than the critical diameter for DLD will follow a zigzag mode, whereas the size of the particles more significant than the required diameter will follow a bumping or displacement mode, which results in separation based on their size differences. It has been demonstrated that DLD pillar arrays with 235 nm nanopillar gaps are capable of separating exosomes ranging in size from 20 to 110 nm 144.

#### 5.1.2. Charge-based techniques

##### 5.1.2.1. Ion-exchange techniques

In order to separate EVs using ion exchange techniques, such as chromatography and metal-affinity systems, an anion exchanger with positively charged functional groups or cations interacts with negatively charged components of EV membranes whose charges are determined by the zeta potential. It is generally possible to release bound EVs by increasing the ionic strength of a buffer by adding high salt concentrations to facilitate the desorption of EVs from positively charged media. Cell culture EVs have been isolated using anion-exchange chromatography (AIEC) in recent years 145.

##### 5.1.2.2. Electrophoresis and dielectrophoresis

A relatively new method for separating EVs by charge is electrophoresis and dielectrophoresis (DEP). It is possible to separate EVs and their subpopulations based on their electrophoretic mobilities in electrophoresis, providing additional information on characteristics of charged EVs that cannot be obtained with size- and density-based approaches. In addition to providing a deeper understanding of both charged and non-charged EVs, the DEP has been successfully implemented in microfluidic systems 146.

##### 5.1.2.3. Ion concentration polarization

By using ion concentration polarization, exosomes can be isolated and preconcentrated simultaneously. Exosomes were concentrated by a factor of 15 every ten minutes using agarose gel and an ion-selective membrane on the chip. As compared to conventional methods, the recovery rate was 60-80%, which is significant 147.

##### 5.1.2.4. Cyclical electrical field-flow fractionation (cyclical ElFFF)

A cyclic ElFFF technique has recently been used to separate exosomes and small and medium-sized EVs from plasma and glioblastoma cells purified from melanoma 148.

#### 5.1.3. Affinity-based techniques

Affinity-based isolation is among the most popular EV isolation techniques, in addition to charge-, density-, and size-based isolation techniques. An affinity-based approach combines a ligand with a protein on an EV membrane, such as a receptor, to achieve highly selective and specific interactions. In order to isolate EVs, antibodies against EV surface proteins are most commonly used as an affinity-based technique. A biotinylated antibody is usually covalently coupled to magnetic beads. Tetraspanin proteins, such as CD9, CD63, and CD81, are commonly enriched on exosome and EV surfaces. Apart from the ability to selectively isolate EVs in general, another major advantage of immunoaffinity is the ability to isolate EVs from different types of cells 149.

#### 5.1.4. Other techniques

Additionally, other techniques have been reported for isolating EVs in addition to those previously mentioned 150.

##### 5.1.4.1. Hydrophobic interaction chromatography (HIC)

By using poly (ethylene terephthalate) (PET) capillary-channeled polymer (C-CP), exosomes have been isolated from exosome-sprinkled human plasma, human urine, and cell line samples. Using PET with weakly ionized surfaces, this technique binds with the hydrophobic surface of exosomes. Gradient elution was used to desorb the exosomes adsorbing on the surface. Due to the presence of other hydrophobic molecules than EVs in a large amount, this technique may not be applicable to all types of samples, including biological fluids 151.

##### 5.1.4.2. Microfluidic platforms

Due to their small size, automation, and minimal sample volume requirements, microfluidic platforms are also among the new emerging techniques for the isolation of EVs. Microfluidic platforms have employed different isolation principles, such as immunocapture. There have also been reports of the use of acoustic trapping microfluidic devices for the isolation of EVs from cell culture media, urine, and plasma 152.

##### 5.1.4.3. Acoustic trapping technology

By using ultrasonic wave scattering, the acoustic trapping technology captures EVs. Acoustic forces trap seeding particles, such as polystyrene beads, with EVs, resulting in clusters of EVs and particles, depending on the particle size, density, and compressibility. Once the ultrasound has been deactivated, the clusters are washed and released. EVs isolated by this method are comparable to those isolated by UC and only require a volume of 12.5 µL 153.

##### 5.1.4.4. Magnetic nanowires

A magnetic nanowire (Fe/Au) has also been used to isolate tumor-derived EVs from cancer cells. The nanowires are internalized into EVs located within a target cell (e.g., cancer cells), and the nanowires are then attracted by a magnetic stand and isolated from the target cell. In comparison with UC and a commercial kit, yield and size distribution were comparable 154.

Due to an increasing interest in EVs research and the shortcomings of conventional ultracentrifugation and precipitation-based isolation techniques for EVs, numerous more advanced methods of EVS isolation have been developed. To obtain high-quality EVs for further analysis in order to determine their properties, functions, cargoes, and potential applications, it is imperative to obtain high-quality EVs for further analysis. 155.

A number of techniques are currently used to fractionate EV subpopulations, such as exomeres and exosomes, including ultracentrifugation, size-exclusion chromatography, ultrafiltration, precipitation, and immunoaffinity capture. An emerging technique is asymmetric flow field-flow fractionation (AsFlFFF/AF4). The use of microfluidics and multistep combined methods is another emerging technique. Since EVs are diverse in origin, complex in nature, and heterogeneous in composition, a combination of methods is often the best option for isolating them. EV isolation and separation technologies should be scalable, more automated, selective to specific subpopulations of EVs, and capable of handling complex biological fluids for therapeutic purposes 156.

### 5.2. MSC-EVs Markers

It is recommended that MSC-EVs might be characterized by at least three positive markers (including one lipid-bound transmembrane protein) and one negative marker. EVs are commonly identified by markers such as tetraspanin families (for example, CD9, CD63, CD81, and CD82), MVB membrane transport (Alix and TSG101), and heat-shock proteins (Hsp70 and Hsp90). CD29, CD44, CD73, CD90, and CD105 are among the markers that MSCs-EVs can express in response to their parental cells. Additionally, this cell-type fingerprint indicates that MSC-EVs have a similar potential to MSCs for treating various diseases, as well as providing targets for characterization 157.

### 5.3. Storage of MSC-EVs

In order to preserve the biological activity of EV during storage, we must be mindful of both the importance and the challenges involved. The storage and formulation of EVs have been the subject of few consistent studies. After 45 days of storage at 80°C or 6 months, the number and size distribution of exosomes as well as their biological activity were not affected 158.

#### 5.3.1. Siliconized vessels

To prevent EVs from adhering to surfaces and from leaking, silicone-lined containers are recommended for EV storage 159.

#### 5.3.2. Phosphate buffered saline

EVs resuspension is commonly performed with phosphate buffered saline. It is recommended to store EVs at -80°C, but it may affect their size, number and function. Studies have shown that EV concentrations remain stable at 4, -20, and -80°C after 1 week of storage. Although EVs aggregate at 4°C, proteins and miRNAs associated with them dramatically decrease at 4°C and -20°C due to storage 159.

#### 5.3.3. NaCl

In order to use EVs products in clinical settings, they need to be suspended in sterile 0.9% NaCl and stored at -80°C. In addition, their morphology and function should be preserved by freezing and thawing rapidly. A single-use formulation of EVs products is recommended since their number decreases after two cycles of freezing and thawing, as well as their morphology and content 160.

#### 5.3.4. Freeze-drying

Research has been conducted on freezing-drying EVs products for long-term storage. It may be cost-effective to store EVs by freeze-drying. Freeze-drying preserves EVs characteristics and functions. In addition, it reduces the cost of transportation 161.

#### 5.3.5. Lyophilization and high dose γ-irradiation

Secretomes derived from peripheral blood mononuclear cells are stable for up to 6 months after lyophilization and high-dose irradiation when stored at -25°C. These lyophilized secretomes might have preserved the bioactivity of samples due to the presence of albumin, cholesterol, and triglycerides 162.

#### 5.3.6. Disaccharide stabilizers

It would be possible to add disaccharide stabilizers to the storage buffer to improve EVs preservation. Among other uses, trehalose is used to cryo-preserve labile proteins in drugs, vaccines, and liposomes. It has been demonstrated that it is safe and tolerable after oral, gastric, and parenteral administration in mice and humans. EVs samples stained with trehalose have been reported to be more stable when stored at -80°C and when lyophilized, as trehalose prevents EVs aggregation and degradation 163.

#### 5.3.7. Mannitol

It is also possible to freeze-dry secretomes for at least two months with mannitol as a cryoprotectant. Furthermore, EV integrity and function can be maintained by adding 5-10% DMSO. A major advantage of lyophilized products is the possibility to develop them off-the-shelf, rather than freezing the parental product for preservation and having to transport it fresh after revitalization and/or expansion, or freezing under stringent conditions. 164.

Furthermore, batch stability must be carefully examined and monitored during storage, regardless of the storage formulation and conditions. In order to assess the stability of the product, it is possible to quantify the particle number, the quantity of total RNA and proteins, as well as the bioactive factor associated with the MoA at different times during storage. 165.

## 6. Mechanism of MSC-EVs in Neurological Diseases

In light of the growing demand for treatment methods for neurodegenerative diseases, stem cells can be used in this area to treat a variety of neurodegenerative disorders 166. The current state of cell-based therapies for neurological disorders must be examined in order to advance the field 167. Since prevailing neurological disorders cannot be cured, medical research should invest in stem cell research and based therapeutic approaches to induce neurological improvements 168. Transplanting functional or healthy cells or products derived from them to the diseased may be an alternative to the development of a cure. MSC-EVs have been found to be highly effective in treating CNS lesions and preventing further damage in some cell-based models 169. A number of bioactive molecules that are essential to the functioning of cells can be packaged inside EVs, thereby protecting them from harsh extracellular environments and allowing them to be safely delivered to the target cells 170. MSCs are ideal candidates for regenerative medicine due to their unique characteristics, such as differentiation, homing, and migration. The immunomodulatory properties of these compounds make them ideal for repairing tissues and organs 171. MSCs have shown clinical potential for the treatment of neurological diseases, kidney diseases, and diabetes in several studies. Numerous researchers have investigated how MSCs benefit the body, but their exact mechanism remains unclear. In the initial hypothesis, paracrine effects were not thought to be responsible for the therapeutic efficacy of MSCs 172. MSC secreted factors may contribute to therapeutic effects both *in vitro* and *in vivo*, according to numerous studies 173. Since the turn of the century, three databases have been developed that provide a comprehensive overview of electric vehicles: EVpedia, ExoCarta, and Vesiclepedia 174. Based on the research conducted so far, it has been concluded that EVs are primarily responsible for transporting cargo between cells via direct fusion with cellular membranes, the endocytic pathway, and the interaction between lipids and receptors.

As an anti-acute brain injury and an anti-Alzheimer's and Parkinson's disease treatment, MSCs have demonstrated satisfactory safety profiles 175. Using umbilical cord blood and bone marrow MSCs has demonstrated significant improvement in multiple sclerosis (MS) 176. Several animal models and clinical trials have shown that a MSC-based treatment can slow the progression of Parkinson's disease as well as overcome its challenges 177. Studies have demonstrated that EV-mediated intercellular correspondence contributes to the removal of waste neuronal and glial material from the nervous system. As well as being able to treat a variety of neurological disorders, this mechanism has opened up a whole new realm of potential applications 178. EVs provide valuable information on CNS functions and disorders since they are crucial to cell-to-cell interactions. It has been suggested that EVs could also be used as drugs delivery vehicles across the BBB, which would treat cells within the central nervous system 179. In spite of the fact that MSC-EVs are derived from a variety of sources and are manipulated to promote beneficial effects in a variety of diseases, they appear to attenuate neurological diseases including Alzheimer's disease, strokes, amyotrophic lateral sclerosis, and Parkinson's disease 180. The characteristics of MSC-EVs make them promising candidates for cell-based therapies.

## 7. Recent MSC-derived EVs for Neurological Diseases

**Figure 4:**
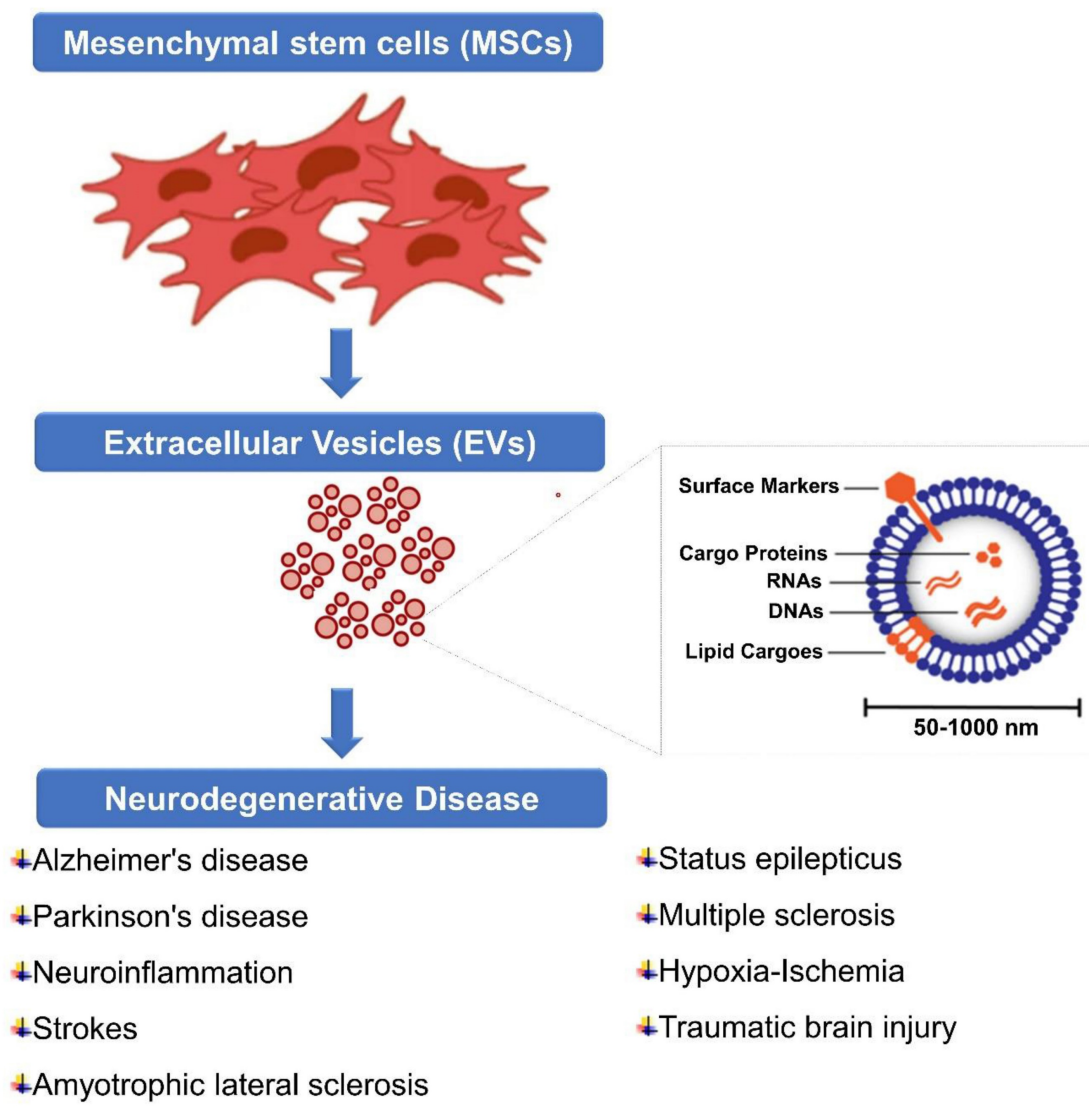
** Emerging therapeutic potential of MSC-derived EVs in neurological diseases.** The therapeutic potential of MSC-derived EVs in neurological diseases has been a subject of growing interest and research. Emerging evidence suggests that MSC-derived EVs hold promise as a novel therapeutic approach for various neurological conditions.

Neurological diseases, such as Alzheimer's disease, Parkinson's disease, stroke, traumatic brain injury, and multiple sclerosis, are characterized by various mechanisms of neuronal damage, inflammation, and impaired tissue repair. MSC-EVs have shown promise in these conditions due to their ability to deliver therapeutic cargo, modulate inflammation, promote tissue repair, and support neuronal survival and function (Figure 4). Here are some ways in which MSC-EVs have been investigated for neurological diseases (Table 1); Stroke and hypoxic-ischemic brain damage are among the neurological diseases that can be modelled in animals and they have been shown to benefit from extracellular vesicles derived from mesenchymal stem cells (MSCs) 181. A study conducted by Wang et al. (2018) examined whether MSC-derived EVs could offer benefits to animals with Alzheimer's disease (AD). A two-week period was followed by the administration of MSC-derived EVs to APP/PS1 mice and their non-transgenic littermates (WT). After that, cognitive behaviours were measured using a novel water maze task and an object recognition task. To assess plasticity of hippocampal synaptic connections, electrophysiological tests were conducted. In primary cultured neurons treated or prepared from APP/PS1 mice, mRNA and protein levels of iNOS were measured by qRT-PCR and western blotting. Treatment with MSC-derived EVs reduces INOS mRNA and protein expression. Cells cultured from APP/PS1 pups were significantly reduced in iNOS mRNA and protein levels by MSC-derived EVs. The MSC-derived EVs improved cognitive behaviour in APP/PS1 mice and rescued synaptic transmission impairments. These results suggest that MSC-derived EVs may suppress the expression of iNOS in a mouse AD model 182.

The mechanism of bone marrow MSC-EVs (BM-MSC-EVs) were studied in a rat model of AD by Sha et al., (2021). Their study was to investigate the cognitive function, the accumulation of amyloid-one (A1-), the deposition of A1-42, and factors related to the decomposition of A1 (NEP and IDE) in rats treated with BM-MSC-EVs. This study found that miR-29c-3p and BACE1 levels in AD and AD models treated with EVs were lower when compared with pre-treatment AD models and when compared with control AD models. It has been predicted and confirmed that miR-29c-3p correlates with BACE1. EV-treated AD neurons were also treated with a DKK1 inhibitor. In AD rats and neurons, BM-MSC-EVs showed therapeutic effects. AD neurons were transported by BM-MSC-EVs with MiR-29c-3p. It has been reported that miR-29c-3p targeted by BACE1. There has been a reduction in the therapeutic efficiency of BM-MSC-EV on AD when miR-29c-3p is silenced in BM-MSCs, which can be reversed by knocking down BACE1 in the BM-MSCs. miR-29c-3p targeted BACE1 and activated the Wnt/β-catenin pathway, and the Wnt/β-catenin pathway inhibition impaired EV therapeutic effects on AD 183.

As they release a highly proactive secretome of soluble factors and EVs, BM-MSCs have been extensively studied for their potential therapeutic role. In addition to direct and indirect amyloid degradation, immunoregulation, and neurotrophic properties, BM-MSC-EVs carry many beneficial characteristics of their parental cells. EVs are very attractive therapeutic options for treating neurodegenerative diseases such as AD. APPswe/PS1dE9 AD mice were injected intracerebrally with BM-MSC-EVs at three and five months of age, a period during which cognitive behavioral abnormalities are not detectable. Cortical and hippocampus dystrophic neurites were reduced in both groups of mice treated with BM-MSC-EVs. Direct degradation of amyloid can be achieved using BM-MSC-EVs that contain Neprilysin. A role for BM-MSC-EVs in AD may be apparent in the early stages, suggesting intervention as soon as overt clinical symptoms appear 184.

AD is characterized by mitochondrial dysfunction. Existing mitophagy inducers, however, are toxic and do not enrich the brain sufficiently 185. Nanosized MSC-derived EVs (MSC-EVs-SHP2) expressing high levels of tyrosine phosphatase-2 (SHP2) have been developed by Xu et al., (2022). SHP2 is efficiently delivered to AD-mice by MSC-EVs-SHP2 because of its high penetration capacity through the blood-brain barrier. Furthermore, NLRP3-activated inflammasomes and mitochondrial damage-associated apoptosis in neuronal cells were significantly reduced by MSC-EVs-SHP2. Mitophagy further decreased apoptosis and inflammation in neurons in AD mouse models. EV-engineering can be used to induce mitophagy in AD patients, providing an effective treatment option 186.

In order to slow down the progression of AD, targeting innate immune cells in the brain could be a promising therapeutic approach due to the role neuroinflammation plays in favoring and accelerating its pathogenesis 187. Accordingly, different AD mouse models have been reported to benefit from MSC-EVs when chronically injected intravenously or intracerebrally. In their study, Losurdo et al., (2019), EVs derived from cytokine-preconditioned MSCs were delivered for the first time intranasally using triple-transgenic mice (3xTg) which might enhance immune modulation and neuroprotection in AD patients. MSC-EVs induced dendritic spine density in the brain and dampened microglia activation. Transgenic mice have been shown to be neuroprotective because of the polarization of primary murine microglia to an anti-inflammatory phenotype *in vitro*. MSC-EVs might be able to be administered in a noninvasive way and demonstrate anti-inflammatory properties, which could enhance their translational potential in AD 187.

The role of MSCs in immune regulation is great, and they provide protective paracrine effects, in part mediated by EVs 198. A number of soluble factors are found in MSC-EVs which are responsible for MSC-EVs being similar to MSCs in terms of their anti-inflammatory and regenerative properties. Cell contact and secretion are both mechanisms through which the MSC modulates microglia activation. Neurodegenerative disorders are mainly caused by inflammation in CNS, caused by microglia cells 199. In a study conducted by Kaniowska et al., (2022), they examined whether MSC-EVs affect how amyloid aggregates activate microglia cells. Their study showed that MSC-EVs prevented proinflammatory mediators from gaining influence, such as tumor necrosis factor (TNF)-α and nitric oxide (NO). Neurodegenerative diseases such as AD, which represent chronic inflammation, upregulate both. MSC-EVs may also be useful for treating neuroinflammatory diseases in the future according to this study 188.

In thrombolysis of ischemic strokes, tissue plasminogen activator (tPA) prevents disruption of the blood-brain barrier (BBB). In conjunction with tPA thrombolysis, MSC-EVs may be able to attenuate hemorrhagic transformation and BBB disruption after ischemic stroke; however, their potential to attenuate these effects is uncertain 169. Qiu et al., (2022) found that aggregation-induced emission luminogens (AIEgens) provide better tracking ability than the commercially available tracer DiR. As a result of passing through the BBB, MSC-EVs were able to accumulate selectively in brain regions of ischemic stroke model mice in real time. In addition, after administration of tPA, astrocytes were able to absorb MSC-EVs more readily. By inhibiting tPA-induced astrocyte activation and inflammation, MSC-EVs also attenuated disruption of the BBB. In addition to blocking NF-kappa B signaling, miR-125b-5p delivered by MSC-EVs also blocks Toll-like receptor 4 (TLR4). In this study, MSC-EVs were found to be noninvasive thrombolytic adjuvants following tPA treatment for ischemic strokes 169.

Cell-free therapy with MSC-EVs for AD is promising mainly because of MSC paracrine activity, however, the exact mechanism is unknown 189. Several studies suggest mitochondrial dysfunction occurs before amyloid plaque formation and neurofibrillary tangle formation in AD, and that it plays an important role in its onset and progression 200. Zhang et al. (2020) conducted an *in vitro* study using a model of Alzheimer's disease (AD) to investigate the potential protective effects of human umbilical cord-derived mesenchymal stem cell-derived extracellular vesicles (hucMSC-EVs) and explore the mechanisms associated with mitochondria. The oxidative stress, mitochondrial function, apoptosis, and AD-related genes in SH-SY5Y cells were assessed after treatment with okadaic acid (OA), followed by treatment with hucMSC-EVs. It was observed that hucMSC-EVs were significantly dephosphorylated at Thr181 (p181-tau), which is elevated in AD. SH-SY5Y cells were also reduced in oxidative stress by hucMSC-EVs after being treated with OA. Furthermore, mitochondrial function and apoptosis resistance were improved in SH-SY5Y cells treated with OA. In a flow cytometric analysis, mitochondria from OA-treated SH-SY5Y cells were partially transferred to SH-SY5Y cells by hucMSC-EVs. Additionally, RNA sequencing has shown that hucMSC-EVs are involved in regulating multiple AD-related genes, signaling pathways, and mitochondrial functions. The results suggest a novel approach for treating AD with MSC-EVs with abundant mitochondria 189.

A neurodegenerative disease without an effective cure, Amyotrophic Lateral Sclerosis (ALS) affects millions around the world 201. A toxic phenotype of astrocytes is associated with ALS, resulting in the death of motoneuron (MN). It is possible to modulate the neurotoxic properties of astrocytes in order to reduce the death rate of MNs. ALS in SOD1^G93A^ mice can be reversed with MSC administration, but the mechanism remains unclear. According to some studies, MSC-secreted EVs cause these effects. An immunohistochemistry study, molecular analysis, and *in vitro* functional analysis were performed by Provenzano et al. in 2022 on astrocytes isolated from symptomatic spinal cords of SOD1^G93A^ mice and their neurotoxicity. ALS patients' inducible neural progenitor cells (iNPCs) are distinguished from their inducible astrocytes. MNs were not neurotoxic to mouse and human ALS astrocytes after exposure to EVs *in vitro*. After exposure to EVs, astrocytes from SOD1^G93A^ showed significant reductions in pathological phenotypes and neuroinflammation. A reduction in reactive oxygen species and increased antioxidant activity were observed in astrocytes when they were exposed to EVs. Nine miRNAs were found to be upregulated in MSC-EVs in a previous study. In this study, single miRNA mimics were transfected into SOD1^G93A^ astrocytes to reduce activation and neuroinflammatory responses. Mapk11 expression is also reduced by miR-466q and miR-467f mimics, while Nrf2 nuclear translocation is increased by miR-466m-5p and miR-466i-3p mimics. MN neurotoxicity was reduced when iAstrocytes were transfected with miR-29b-3p mimics. Astrocytes' reactive phenotype and neurotoxicity are modulated by MSC-EVs' anti-inflammatory and antioxidant-shuttled miRNAs, which represents a therapeutic strategy in ALS 190.

Diseases associated with aging are often caused by inflammation. Microglial activation causes neuronal cell death in AD caused by amyloidbeta oligomers 202. Inflammatory diseases can be treated using stem cells since they are paracrine and capable of responding to an inflammatory environment. Neurological recovery promoted by stem cells, however, is poorly understood 203. These mechanisms were revealed by Markoutsa et al., (2021) through the combination of MSCs and lipopolysaccharide or amyloidbeta activated microglia may result in the generation of MSC-derived secretomes. After that, EVs secreted from MSCs and non-MSCs were compared for their immunomodulatory effects. A comparison between EVs from MSC and those from non-MSC found that EVs from MSC inhibited microglia and astrocyte activation, amyloid deposition, demyelination, memory loss, and anxiety-like behavior more than non-MSC EVs. MSC-EVs were found to be upregulated by at least 19 microRNAs (miRNAs). Based on KEGG pathway analysis, the overexpressed miRNAs target genes involved in the signaling pathway associated with the toll-like receptor-4 (TLR4). MiRNAs released by MSC enhanced immunity regulation when combined with activation microglia secretomes 191.

Age decreases the brain's regeneration capacity. The ability to function may be affected by both brain damage and neurodegenerative diseases. It is possible to isolate MSCs, a type of adult stem cell, from a variety of adult tissues. EVs and secretomes derived from MSCs have been investigated for their therapeutic potential. A study by Chen et al., (2019) found that blocking the prostaglandin E2 receptor 4 pathway in MSCs resulted in higher EV release and sorted proteins, including anti-inflammatory cytokines, factors that affect astrocyte function, BBB integrity, and microglial migration. This study observed significant improvements in cognitive function, learning abilities, and memory. These improvements were observed when researchers induced extracellular vesicles derived from mesenchymal stem cells (MSC-EVs) using EP4 antagonists. Additionally, the study found that the induced MSC-EVs inhibited reactive astrogliosis, reduced inflammation, decreased microglial infiltration into damaged hippocampal tissues, and strengthened the blood-brain barrier 204.

The benefits of MSC-EVs have been attributed to the isolation of MSCs from a range of human tissues for use in therapy. It has become obvious that MSCs from every tissue type possess unique properties despite the fact that they share a number of cardinal stem cell characteristics. It is essential to understand the unique characteristics of MSCs and MSC-EVs from different tissues in order to develop effective stem cell therapies 205. Using comprehensive gene expression databases and sophisticated analytical tools, transcriptomic approaches can be used to analyze these properties. A study was conducted by Terunuma et al., (2021) examining the transcriptomes of dental pulp and adipose tissue MSC-EVs. Comparing MSC-EV transcriptomes with those of cellular MSCs was also performed. To culture MSCs, an adipose tissue specimen and a dental pulp specimen were used. Next-generation sequencing was used to analyze transcriptomic data from conditioned culture media prepared for MSC-EVs. Results shown that, transcriptomic analysis of MSC-EVs (dental pulp-derived MSCs) demonstrated distinct transcriptomic signatures of neurogenesis and neural retinal development. The transcriptional signatures of mitochondrial activity and skeletal development were distinct in MSC-EVs (adipose tissue-derived MSCs). Consequently, dental pulp-derived MSC-EVs may be useful therapeutic targets for neurodegenerative disorders and retinal diseases. The skin and muscles can be rejuvenated with MSC-EVs from adipose tissue. Developing new therapeutic targets for MSC-EVs may be made easier with a better understanding of MSC-EVs from diverse tissue types 206.

Neurodegeneration, inflammation, altered neurogenesis, and cognitive and memory deficits are all symptoms of a disordered hippocampus caused by status epilepticus (SE). Intranasally (IN) administered EVs from bone marrow-derived MSCs were examined for their impact on SE-induced adverse effects. Pilocarpine-induced SE for 2 hours was followed by administration of MSC-EVs IN over the next 24 hours. Six hours after administration, MSC-EVs reached the hippocampus and decreased glutamatergic and GABAergic neuron loss and inflammation. Further, MSC-EVs preserved hippocampal neurogenesis and cognitive and memory function for long periods by being neuroprotective and antiinflammatory, while animals receiving vehicles demonstrated diminished neurogenesis, persistent inflammation, and functional impairment. MSC-EV administration helps prevent SE-induced memory impairment and reduces cognitive impairment due to SE in the hippocampus, according to these results 192.

One of the leading causes of blindness is loss of retinal ganglion cells (RGC) and their axons, in addition to traumatic (optic neuropathy) and degenerative (glaucoma) eye diseases 207. It has been shown that MSCs can function as both neuroprotectors and axogens on RGCs in both models. Studies have demonstrated that MSC-EVs can deliver proteins, mRNAs, and miRNAs. EVs derived from bone marrow-derived MSCs (BMSCs) were isolated and tested in a rat model of optic nerve crush (ONC). The effects of BMSC-EVs on retinal primary cultures were significantly neuroprotective and neurogenic. After the ONC injections and weekly intravitreal exosome injections, optical coherence tomography, electroretinography, and immunohistochemistry were performed. As a result of using BMSC-EVs, RGC axons survived and regenerated better, while RGC axon losses and dysfunction were partially prevented. EVs derived from BMSCs carrying cargo did not reach inner retinal layers when Argonaute-2, a crucial miRNA effector molecule, was knocked down. It seems that BMSC-EVs can be used for the treatment of traumatic and degenerative ocular diseases without relying on cells 208.

Multiple sclerosis (MS) and inflammation-mediated demyelinating diseases are mediated by EVs, which are important mediators of intercellular communication. As part of a study conducted by Laso-Garca et al. (2018), a human adipose tissue-derived MSC-EVs were administered intravenously as a treatment for Multiple Sclerosis in animals induced with Theiler's murine encephalomyelitis virus (TMEV). As soon as the disease had been established, EVs were administered to SJL/J mice. By administering EVs intravenously to mice infected with TMEV, motor deficits were improved, brain atrophy was reduced, subventricular zone proliferation increased, and spinal cord inflammation was decreased. The treatment with EVs also reduced Iba-1 staining and glial fibrillary acidic protein expression in the brain. Microglia are also shown to be modulated by EVs in addition to their activation state. A significant reduction in plasma cytokine levels is observed in TMEV mice treated with EVs, mainly in Th1 and Th17 phenotypes, confirming EVs' immunomodulatory potential. The effects of EV administration on motor deficits were observed as a result of reduced brain atrophy and promoting remyelination through immunomodulatory effects. A further investigation of EVs as possible treatment for MS's neurodegenerative phase is needed 193.

Inflammatory/neurodegenerative diseases, such as MS and ALS, benefit from the neuroprotective, immunomodulatory, and neuroregenerative properties of MSCs. By releasing EVs carrying proteins, mRNAs, and microRNAs (miRNAs), MSCs exert their immunomodulatory effects by targeting cells to alter their function. Giunti et al., (2021) identified 9 miRNAs that were dramatically downregulated in IFN-γ-primed MSCs, however, at various levels in EVs which are derived from them. In activated N9 microglia cells, as well as in primary microglia isolated from ALS-prone SOD1^G93A^ mice, miR-467f and miR-466q have been shown to downregulate TNF and Il1b expression. Microglia are suppressed in their pro-inflammatory state by miR-467f and miR-466q, which inhibit the p38 MAPK pathway through inhibition of the expression of the genes they target, Map3k8 and Mk2. Furthermore, this study demonstrated that mice suffering from experimental autoimmune encephalitis (EAE) whose spinal cord is administered s-EV showed decreased expression of neuroinflammation markers. This study suggests that MSC-EVs manipulate neuroinflammation by modulating the immune response mediated by microglia 194.

Bioactive factors released by MSCs can modulate the immune system and promote regeneration. There are many characteristics of MS, such as inflammation, demyelination, gliosis, and axonal loss. A murine model of MS was developed by Clark et al. (2019), in which animals were treated with saline, placenta-derived MSCs, and PMSC-EVs at low and high doses at the onset of the disease. PMSC-EV and high-dose PMSC-EV-treated animals showed superior results to saline-treated animals. Symptoms of mice treated with PMSCs and PMSC-EVs improved as DNA damage was reduced in oligodendroglial cells and myelination increased. Using PMSC-EVs *in vitro*, it has been demonstrated that they stimulate the differentiation of endogenous oligodendrocyte precursors into mature myelinating oligodendrocytes to promote myelin regeneration. According to these findings, PMSCs secrete EVs as part of their mechanism of action. EVs from PMSCs have shown potential in animal models of MS as an alternative to cell-based therapies 195.

Neonatal encephalopathy caused by hypoxia-ischemia (HI) is a major contributor to childhood mortality. The brain's microenvironment and MSCs interact unexpectedly during neonatal HI, altering therapeutic efficacy. The majority of MSCs' therapeutic effects are thought to be mediated by EVs in a paracrine manner, suggesting that cell therapy might not be the best option 209. Kaminski et al., (2020) examined the impact of MSC-EVs on neonatal brain injury associated with hyperoxia. As a result of HI-induced damage to the striatum, MSC-EVs significantly reduced micro- and astroglial activation. A significant reduction in TNFa expression occurs when MSC-EVs are used, while an increase in YM-1 and TGFb expression occurs when MSC-EVs are used. In addition to decreasing astrocytic expression of C3, MSC-EVs also increased neurotrophic factors (BDNF, VEGF, and EGF) in astrocytes. There was an increase in neural density and vessel density in the neurogenic region adjacent to the striatum, along with proliferating cells. In addition to improving oligodendrocyte maturation and myelination, MSC-EV-mediated neuroprotection improved oligodendrocyte function. This study demonstrated that MSC-EVs improve key developmental processes in the neonatal brain by providing anti-inflammatory effects, promoting regenerative responses, and improving regenerative responses. HI-induced secondary brain damage can be prevented by MSC-EVs through different cellular target mechanisms. Neonatal brain injury may benefit from MSC-EV treatment instead of risk-associated cell therapies 196.

A retinal ganglion cell (RGC) degeneration leads to blindness due to retinal and/or optic nerve injury. In order to suppress this neurodegeneration, extensive efforts have been made. There was a significant neuroprotective and axogenic effect of MSCs derived from somatic tissue on RGCs 210. Alternative sources of MSCs include human embryonic stem cells (ES-MSCs), which proliferate faster, express lower levels of inflammatory cytokines, and modulate the immune system. Human ES-MSC EVs have not yet been evaluated for their potential therapeutic effects and underlying mechanisms on optic nerve injury. Seyedrazizadeh et al., (2020) injected human ES-MSC EVs to an optic nerve crush (ONC) mouse model. On two months after treatment, immunohistofluorescence, reverse and antegrade tracings of RGCs, Western blots, tauopathy of RGCs, and function assessments were used to assess ONC improvement and the underlying mechanisms of human ES-MSC EVs. RGC survival and retrograde and anterograde tracing of Brn3a+ RGCs were significantly improved in the ES-MSC EVs compared with the vehicle group. The EVs also significantly increased GAP43+ axon counts in the optic nerve and improved cognitive visual behaviour. Furthermore, cis-p-tau is detected in injured RGCs after ONCs show signs of tauopathy in the RGCs at the beginning of the disease, which is a key mediator of neurodegeneration. EV treatment significantly reduced cis p-tau expression. It has been speculated that human ES-MSC EVs could be used as an off-the-shelf and cell-free product to treat injured RGCs and degenerative eye diseases. In addition, human ES-MSC EV may be able to restore RGC function by rescuing tauopathy 211.

Researchers used differential ultracentrifugation to test the therapeutic effects of human bone-marrow derived MSCs in three-dimensional (3D) cell cultures 212. Over the course of four months, non-transgenic (NT) and 5XFAD mice were given small EVs intranasally (IN). Various behavioural tests were performed on mice to measure learning and memory changes. GFAP and amyloid beta levels were analyzed by immunohistochemistry in brain slices. There was no significant difference between hMSC-EVs treated 5XFAD mice and NT mice in cognitive tests. hMSC-EVs treated 5XFAD mice performed significantly better than those treated with saline. Hippocampus plaque loads were also reduced in mice treated with hMSC-EV. Additionally, mice treated with hMSC-EVs showed less colocalization of GFAP on plaque surfaces than mice treated with saline. As a result of these studies, it appears that IN administration of MSC EVs could have a beneficial effect on AD progression 197.

The robust anti-inflammatory and neuroprotective properties of EVs derived from hMSCs make them particularly promising as biologics for treating neurological and neurodegenerative conditions as a result of their strong anti-inflammatory properties 213. Intranasal (IN) injections of EVs have also gained attention due to their noninvasive nature, their capability of repeated administration, and the ability to penetrate multiple forebrain regions quickly 213. A lack of injury-induced signals is not believed to influence IN-administered EV entry into different brain regions. Kodali et al., (2020) examined the distribution of IN-administered hMSC EVs in neurons and microglia of intact and status epilepticus (SE) injured rats. EVs labeled with PKH26 were unilaterally injected into the left nostrils of naive rats and rats exposed to kainate-induced SE for two hours. In multiple forebrain regions, PKH26 + EVs were quantified six hours later by performing serial brain sections and examining them under confocal microscopy. Forebrains that were intact and those that were SE-injured showed an incredible presence of EVs bilaterally. Nearly all regions of the forebrain included EVs in a comparable percentage. EVs, however, were more prevalent in hippocampal CA1 subfields and entorhinal cortex neurons of SE-affected animals. On the other hand, microglia were highly comparable in their incorporation of EVs throughout the forebrain. It is effective to deliver EVs bilaterally to neurons and microglia in the injured forebrain when the EVs are administered unilaterally through IN. Brain injury areas show higher EVs incorporation, suggesting that injury-related signals may play a role in enabling EVs to target neurons, which may be useful as EVs therapy for neurodegenerative diseases like traumatic brain injury, stroke, MS, and AD 214.

## 8. Clinical applications of MSC-EVs plays in neurodegenerative diseases

The composition of EVs includes a variety of different types of cargos, such as nucleic acids, proteins, and metabolites. Native EVs and their cargos have been studied initially for their regulatory and therapeutic effects. EVs play an important role in maintaining and recovering neural function in the nervous system as important mediators of intercellular communication 215. As nanotechnology develops rapidly, enhanced therapeutic capabilities have been developed for EVs, including targeted drug delivery. Clinical approval and marketing authorization have been obtained for some well-established synthetic nanoparticles, such as liposomes, the first nano-drug delivery systems. The rapid clearance of these agents from the blood and the activation of the innate immune system limits their therapeutic potential. EVs are more complex and biocompatible than synthetic drug delivery systems and have lower immunogenicity than liposomes 216. EVs are functionally pharmacokinetically related proteins that contribute to their large volume of biodistribution and higher retention in circulation. EVs' surface molecules enable them to cross the blood-brain barrier (BBB), deliver their cargos, and evoke responses in recipient cells. As a result of these characteristics, EVs have considerable therapeutic potential in the treatment of disorders of the central nervous system (CNS). 217.

A number of pharmaceutical companies as well as the academic community have expressed interest in EV-based therapeutics. The number of clinical trials for EV-based therapeutics in humans is rapidly increasing, and more are currently being conducted 218. The following clinical trials are currently registered in the field of neurological disorders: NCT04388982 (Alzheimer's disease), NCT03384433 (cerebrovascular disorders), and NCT05490173 (neurodevelopmental disorders). In all three trials, MSCs provided EVs, although their tissue sources differed. In NCT04388982 (Alzheimer's disease), allogenic adipose MSCs were applied, whereas in NCT03384433 (cerebrovascular disorder), allogenic bone marrow MSCs were applied. It should be noted that NCT03384433 (cerebrovascular disorders) used allogenic MSCs transfected with miR-124, which may provide evidence for the efficacy of EV-based gene therapies in humans. Specifically, in NCT05490173 (neurodevelopmental disorders) and NCT04388982 (Alzheimer's disease), EVs were administered intranasally, while in NCT03384433 (cerebrovascular disorders), EVs were administered intravenously. There will be exciting results from these representative clinical trials for EV-based therapeutics in neurodegenerative diseases in humans 215.

## 9. Limitation of MSC-EVs therapeutics

MSC therapeutics still have additional challenges, despite engineered MSC sources being able to overcome specific limitations. Immortalized live cell therapies may not have the same engraftment properties as normal cells, since they may be immunogenic and tumorigenic 219. It has been reported that embryonic stem cells can overcome some of the limitations associated with parental cells, including safety, reproducibility, and cost-effectiveness in terms of storage and maintenance. The use of engineered EVs for clinical purposes may be a promising new therapeutic approach. In addition, to overcome current obstacles in the development of EVs-based therapeutics, it is necessary to standardize and optimize the production of EVs, and further research is required to better understand their mechanisms 220.

There are several advantages to using EVs obtained from naive stem cells for patients with brain disorders, although the use of EVs obtained from naive stem cells for patients with brain diseases does have some limitations. 175. As a result of the use of different donors with different tissues, donor heterogeneity is one of the main obstacles preventing the application of MSCs and EV therapeutics in clinical settings. It is important to note that independent MSC-EV preparations may differ in therapeutic potential based on their donor age, comorbidities (obesity and disease condition), artificial niches of MSCs (preconditioning or external stimuli), culture methods, and culture conditions. A robust quality control for each EV production lot should be conducted in addition to developing a production method that minimizes donor-to-donor and batch-to-batch variations 221.

Despite the potential of native EVs for treating brain diseases, their clinical application is limited by their short half-life, limited targeting, rapid clearance after application, and inadequate payload. 222. The BBB can be crossed by native EVs under conditions similar to stroke-like inflammation, however, whether they are able to cross intact BBB remains to be determined. Both preclinical and human studies have shown that blood levels of EVs decrease rapidly after systemic administration of EVs, and EVs accumulate in the lung, liver, and spleen until approximately 10 days following the administration of EVs. Furthermore, macrophage/microglial clearance shortens the circulation time of EVs 223.

### 9.1. Limitation can be solved by some modification methods

Modification methods offer potential solutions to overcome certain disadvantages associated with extracellular vesicles derived from mesenchymal stem cells (MSCs) in the treatment of neurodegenerative diseases. These modifications can help optimize their therapeutic properties and improve their efficacy. By implementing suitable modification approaches, the limitations of MSC-derived extracellular vesicles can be mitigated, leading to enhanced therapeutic outcomes in the treatment of neurodegenerative diseases 224.

**9.1.1. Limited Stability and Half-Life:** Extracellular vesicles may have a short half-life and limited stability, which can reduce their effectiveness. Modification methods such as genetic engineering or surface coating with stabilizing agents can enhance their stability and prolong their circulation time 225.

**9.1.2. Targeting Specific Cell Types:** Extracellular vesicles derived from MSCs may have a non-specific uptake by cells, limiting their ability to specifically target affected cells in neurodegenerative diseases. Modification techniques such as surface modification with targeting ligands or engineering vesicles to carry specific receptors can improve their targeting capability 226.

**9.1.3. Cargo Loading Efficiency:** Efficient loading of therapeutic cargo, such as miRNAs or proteins, into extracellular vesicles can be challenging. Modification approaches such as electroporation, sonication, or exogenous loading methods can enhance the cargo loading efficiency, ensuring effective delivery of therapeutic molecules 227.

**9.1.4. Immune Response:** The immune response triggered by extracellular vesicles can limit their therapeutic efficacy. Modification methods such as membrane engineering or removal of immunogenic components can mitigate immune reactions and improve the vesicles' immunomodulatory properties 228.

**9.1.5. Low Yield:** The yield of extracellular vesicles from MSCs may be limited, making it difficult to obtain sufficient quantities for therapeutic purposes. Modification strategies such as optimizing cell culture conditions, genetic modification, or exosome isolation techniques can improve the yield of extracellular vesicles 229. By employing modification methods tailored to address these challenges, the limitations associated with extracellular vesicles derived from MSCs in the treatment of neurodegenerative diseases can be overcome. These modifications can enhance their stability, targeting specificity, cargo loading efficiency, immune compatibility, and yield, thereby improving their therapeutic potential in neurodegenerative disease management.

## 10. Future perspectives

Recent advancements in the treatment of neurodegenerative diseases involve the utilization of extracellular vesicles (EVs) derived from mesenchymal stem cells (MSCs). These EVs are small, membrane-bound structures released by MSCs that contain various bioactive molecules, such as proteins, nucleic acids, and lipids. Researchers have discovered that these EVs possess therapeutic properties and can exert beneficial effects on the nervous system. One of the key advantages of using EVs for neurodegenerative disease treatment is their ability to cross the blood-brain barrier, which allows them to reach the affected areas of the brain. EVs derived from MSCs have been shown to enhance neuronal survival, promote neurogenesis (the formation of new neurons), and modulate neuroinflammation, thereby reducing the progression of neurodegenerative diseases. Additionally, these EVs can transfer specific molecules, such as microRNAs, which play crucial roles in regulating gene expression. By delivering these microRNAs to target cells in the brain, EVs can modulate gene expression patterns and promote neuroprotection. Moreover, EVs derived from MSCs can also act as carriers for therapeutic drugs or molecules, allowing targeted delivery to the affected brain regions. This targeted approach minimizes potential side effects and enhances the therapeutic efficacy of the delivered substances. Researchers are actively exploring the potential of EV-based therapies for various neurodegenerative diseases, including Alzheimer's disease, Parkinson's disease, and amyotrophic lateral sclerosis (ALS). Preclinical studies have demonstrated promising results, showing improvements in disease-related symptoms and pathological changes. In summary, the use of EVs derived from MSCs represents a novel and promising strategy for treating neurodegenerative diseases. These EVs possess therapeutic properties, can cross the blood-brain barrier, transfer bioactive molecules, and act as targeted drug carriers. Further research and clinical trials are needed to fully understand and harness the potential of this approach in clinical settings.

A potential therapeutic application for these vesicles is currently under investigation in the EV field. Neurological conditions, such as Parkinson's disease, are particularly challenging to treat with this approach 230. Tests have been conducted on therapeutic cargo delivered on EVs and on EVs released with pathogenic protein forms associated with neurodegenerative diseases. The number of EVs in neurological diseases may be upregulated or downregulated; it would be important to understand which neuronal subtypes release EVs associated with neurological diseases 105. In a transgenic mouse model of Alzheimer's disease, researchers showed that reducing EVs release using a neutral sphingomyelinase inhibitor (GW4869) altered the animals' neuropathology, suggesting that reducing EVs release could be therapeutic 231. Due to the potential side effects associated with reducing EVs numbers, specificity may be required to minimize them (for example, targeting distinct pathways or neurons).

The use of extracellular vesicles (EVs) derived from mesenchymal stem cells (MSCs) has gained significant interest in the field of regenerative medicine. MSC-EVs are small membrane-bound vesicles released by MSCs that contain a variety of bioactive molecules, including proteins, nucleic acids, and lipids. Here are some future perspectives of MSC-EV therapies:

1. Targeted Delivery: MSC-EVs can be engineered to carry specific therapeutic cargo, such as small interfering RNA (siRNA), microRNA, or growth factors. These cargo-loaded MSC-EVs can be designed to target specific cells or tissues affected by neurodegenerative diseases. By encapsulating therapeutic molecules within EVs, it may enhance their stability, protect them from degradation, and improve their delivery to the intended site of action.

2. Immunomodulation: MSC-EVs possess immunomodulatory properties similar to their parent cells. They can modulate immune responses, regulate inflammation, and promote tissue repair. MSC-EVs have the potential to be used as a cell-free alternative to MSC transplantation, providing immunomodulatory effects without the risks associated with cell-based therapies.

3. Combination Therapies: MSC-EVs can be used in combination with other therapies to enhance their effectiveness. For example, MSC-EVs can be co-administered with other neuroprotective agents or used alongside gene therapy approaches. This synergistic combination may provide additive or even synergistic effects, improving the outcomes of treatment.

4. Biomarkers and Diagnostics: MSC-EVs carry specific molecules that reflect the physiological and pathological state of their parent cells. Analysis of MSC-EVs' cargo can provide valuable insights into the underlying disease processes, serving as potential biomarkers for diagnosing and monitoring neurodegenerative diseases. This could lead to the development of non-invasive diagnostic tools and personalized treatment strategies.

Regulatory Approval and Clinical Translation: While MSC-EV therapies hold great promise, there are still challenges to overcome before they can be widely implemented in clinical settings. Standardization of isolation methods, characterization techniques, and quality control measures for MSC-EVs are necessary for regulatory approval. Additionally, large-scale production methods need to be established to meet the demands of clinical applications.

Overall, MSC-EV therapies have significant potential in the field of regenerative medicine and neurodegenerative diseases. Further research and development are needed to optimize their therapeutic potential, establish safety and efficacy profiles, and pave the way for their clinical translation.

## 11. Conclusion

There has been increasing evidence that EVs have a role to play within the brain and central nervous system (both in healthy and diseased conditions), due to the growing evidence that they remove unwanted material and provide intercellular communication between cells. A common pathogenic mechanism is suggested by the possibility that EVs propagate misfolded proteins associated with neurodegenerative disorders (e.g., prion disease). It has not yet been demonstrated in human disease through formal experiments, most of which have been performed in cell culture and transgenic mouse models. Neurological diseases can be diagnosed by measuring the amount of protein or genetic material present in extracellular vesicles (e.g., miRNA expression) found in peripheral tissues or the central nervous system. There is still a need to standardize methodologies and test across different cohorts to fully realize the potential of EVs for neurological disease diagnosis. The ability to package active EVs or specifically target cargo will also assist in determining whether they participate in interneuronal communication. Additionally, the BBB facilitates the movement of neuronal EVs within and between the peripheral nervous system. Studying these factors mechanistically will be necessary to determine whether EVs are causative or the result of disease processes, rather than correlating them with other factors.

## Figures and Tables

**Table 1:** Some MSC derived EVs as potential therapy for neurodegenerative diseases

Source of MSC-EVs	Disease / Conditions	*In vitro* model	*In vivo* model	Molecular mechanism	Therapeutic application	References
Bone marrow	AD		APP/PS1 mice and their non-transgenic littermates	EVs from MSCs affected iNOS expression by lowering Aβ levels	Enhanced synaptic plasticity and cognitive performance in a mouse model of AD.	182
bone marrow	AD		AD Rat model	BACE1 was targeted by miR-29c-3p, which activated the Wnt/β-catenin pathway	Wnt/β-catenin pathway inhibition impaired EV therapeutic effects on AD	183
bone marrow	AD		APPswe/PS1dE9 AD mice	BM-MSC-EVs are effective at reducing the Aβ plaque burden and the amount of dystrophic neurites in both the cortex and hippocampus.	The presence of Neprilysin on BM-MSC-EVs, opens the possibility of a direct β-amyloid degrading action.	184
bone marrow	AD		C57BL/6 mice	NLRP3-activated inflammasomes and mitochondrial damage-associated apoptosis in neuronal cells were significantly reduced by MSC-EVs-SHP2	EV-engineering can be used to induce mitophagy in AD patients, providing an effective treatment option	186
bone marrow	AD	Microglial cells	Triple-transgenic AD mice (3xTg-AD)	MSC-EVs induced dendritic spine density in the brain and dampened microglia activation.	MSC-EVs might be able to be administered in a non-invasive way and demonstrate anti-inflammatory properties, which could enhance their translational potential in AD	187
bone marrow	AD	Microglial cells		MSC-EVs prevented proinflammatory mediators from gaining influence, such as tumor necrosis factor (TNF)-α and nitric oxide (NO).	The use of MSC-EVs as a promising therapeutic tool to treat neuroinflammatory diseases	188
Human umbilical cord	Ischemic stroke	bend.3 cell line	C57BL/6 N mice	By inhibiting tPA-induced astrocyte activation and inflammation, MSC-EVs also attenuated disruption of the BBB.	MSC-EVs were found to be non-invasive thrombolytic adjuvants following tPA treatment for ischemic strokes	169
Human umbilical cord	AD	SH-SY5Y cells		hucMSC-EVs were significantly dephosphorylated at Thr181 (p181-tau), which is elevated in AD. SH-SY5Y cells were also reduced in oxidative stress by hucMSC-EVs after being treated with OA	Novel approach for treating AD with MSC-EVs with abundant mitochondria	189
bone marrow	ALS	Mouse SOD1G93A astrocytes and iAstrocytes	B6SJL-TgN SOD1/G93A1Gur mice	Argocytes' reactive phenotype and neurotoxicity are modulated by MSC-EVs' anti-inflammatory and antioxidant-shuttled miRNAs, which represents a therapeutic strategy in ALS	MSC-EVs may be therapeutically effective across different subtypes of ALS, beyond SOD1 as a model.	190
hMSCs	AD	HMC3 cells	Male C57BL/6 mice	EVs from MSC inhibited microglia and astrocyte activation, amyloid deposition, demyelination, memory loss, and anxiety-like behavior more than non-MSC EVs.	MiRNAs released by MSC enhanced immunity regulation when combined with activation microglia secretomes	191
bone marrow	Status epilepticus (SE)		Male C57BL/6J mice	MSC-EVs reached the hippocampus and decreased glutamatergic and GABAergic neuron loss and inflammation	MSC-EV administration helps prevent SE-induced memory impairment and reduces cognitive impairment due to SE in the hippocampus, according to these results	192
Human ADMSCs	Multiple sclerosis		Female SJL/J mice	A significant reduction in plasma cytokine levels is observed in TMEV mice treated with EVs, mainly in Th1 and Th17 phenotypes, confirming EVs' immunomodulatory potential	he effects of EV administration on motor deficits were observed as a result of reduced brain atrophy and promoting remyelination through immunomodulatory effects	193
bone marrow	Neuroinflammation	Murine microglial cell line N9	B6SJL-TgN SOD1/G93A1Gur mice	In activated N9 microglia cells, as well as in primary microglia isolated from ALS-prone SOD1G93A mice, miR-467f and miR-466q have been shown to downregulate TNF and Il1b expression.	MSC-EVs manipulate neuroinflammation by modulating the immune response mediated by microglia	194
chorionic villus tissue	MS	SH-SY5Y neuroblastoma cell line	C57BL/6J mice	MSC-EVs stimulate the differentiation of endogenous oligodendrocyte precursors into mature myelinating oligodendrocytes to promote myelin regeneration	They have shown potential in animal models of MS as an alternative to cell-based therapies	195
bone marrow	hypoxia-ischemia (HI)		C57BL/6 mice	A significant reduction in TNFa expression occurs when MSC-EVs are used, while an increase in YM-1 and TGFb expression occurs when MSC-EVs are used.	Neonatal brain injury may benefit from MSC-EV treatment instead of risk-associated cell therapies	196
bone marrow	AD		5XFAD mice and NT mice	hMSC-EVs treated 5XFAD mice performed significantly better than those treated with saline. Hippocampus plaque loads were also reduced in mice treated with hMSC-EV	MSC EVs could have a beneficial effect on AD progression	197

## References

[1] Holbrook JA, Jarosz-Griffiths HH, Caseley E, Lara-Reyna S, Poulter JA, Williams-Gray CH (2021). Neurodegenerative disease and the NLRP3 inflammasome. Front Pharmacol.

[2] Faden AI, Barrett JP, Stoica BA, Henry RJ (2021). Bidirectional brain-systemic interactions and outcomes after TBI. Trends Neurosci.

[3] Feigin VL, Vos T, Nichols E, Owolabi MO, Carroll WM, Dichgans M (2020). The global burden of neurological disorders: translating evidence into policy. Lancet Neurol.

[4] Khachaturian A, Dengel A, Dočkal V, Hroboň P, Tolar M (2023). Accelerating Innovations for Enhanced Brain Health. Can Artificial Intelligence Advance New Pathways for Drug Discovery for Alzheimer's and other Neurodegenerative Disorders? J Prev Alzheimers Dis.

[5] Lamptey RN, Chaulagain B, Trivedi R, Gothwal A, Layek B, Singh J (2022). A review of the common neurodegenerative disorders: current therapeutic approaches and the potential role of nanotherapeutics. Int J Mol Sci.

[6] Ullah MF, Ahmad A, Bhat SH, Abu-Duhier FM, Barreto GE, Ashraf GM (2019). Impact of sex differences and gender specificity on behavioral characteristics and pathophysiology of neurodegenerative disorders. Neurosci Biobehav Rev.

[7] Gazerani P (2023). Contribution of Central and Peripheral Glial Cells in the Development and Persistence of Itch: Therapeutic Implication of Glial Modulation. Neuroglia.

[8] Weigel M, Wang L, Fu Mm (2021). Microtubule organization and dynamics in oligodendrocytes, astrocytes, and microglia. Dev Neurobiol.

[9] Yu W, Ying J, Wang X, Liu X, Zhao T, Yoon S (2021). The involvement of lactosylceramide in central nervous system inflammation related to neurodegenerative disease. Front Aging Neurosci.

[10] Jiang X, Li S, Feng X, Li L, Hao J, Wang D (2022). Mushroom polysaccharides as potential candidates for alleviating neurodegenerative diseases. Nutrients.

[11] Scarfò G, Piccarducci R, Daniele S, Franzoni F, Martini C (2022). Exploring the Role of Lipid-Binding Proteins and Oxidative Stress in Neurodegenerative Disorders: A Focus on the Neuroprotective Effects of Nutraceutical Supplementation and Physical Exercise. Antioxidants.

[12] Kwon HS, Koh S-H (2020). Neuroinflammation in neurodegenerative disorders: The roles of microglia and astrocytes. Transl Neurodegener.

[13] Li K, Li J, Zheng J, Qin S (2019). Reactive astrocytes in neurodegenerative diseases. Aging Dis.

[14] Taylor X, Cisternas P, Jury N, Martinez P, Huang X, You Y (2022). Activated endothelial cells induce a distinct type of astrocytic reactivity. Commun Biol.

[15] Radenovic L, Nenadic M, Ułamek-Kozioł M, Januszewski S, Czuczwar SJ, Andjus PR (2020). Heterogeneity in brain distribution of activated microglia and astrocytes in a rat ischemic model of Alzheimer's disease after 2 years of survival. Aging.

[16] Parhizkar S, Holtzman DM (2022). APOE mediated neuroinflammation and neurodegeneration in Alzheimer's disease. Seminars in immunology: Elsevier.

[17] Olah M, Menon V, Habib N, Taga MF, Ma Y, Yung CJ (2020). Single cell RNA sequencing of human microglia uncovers a subset associated with Alzheimer's disease. Nat Commun.

[18] Nicholson M, Wood RJ, Gonsalvez DG, Hannan AJ, Fletcher JL, Xiao J (2022). Remodelling of myelinated axons and oligodendrocyte differentiation is stimulated by environmental enrichment in the young adult brain. Eur J Neurosci.

[19] Seth B, Yadav A, Tandon A, Shankar J, Chaturvedi RK (2019). Carbofuran hampers oligodendrocytes development leading to impaired myelination in the hippocampus of rat brain. Neurotoxicology.

[20] Shang K, Cheng C, Qin C, Xiao J, Deng G, Bu B-T (2021). Case Report: Clinical and Imaging Characteristics of a Patient with Anti-flotillin Autoantibodies: Neuromyelitis Optica or Multiple Sclerosis?. Front Immunol.

[21] Dimovasili C, Fair AE, Garza IR, Batterman KV, Mortazavi F, Moore TL (2022). Aging compromises oligodendrocyte precursor cell maturation and efficient remyelination in the monkey brain. GeroScience.

[22] Baloni P, Funk CC, Readhead B, Price ND (2021). Systems modeling of metabolic dysregulation in neurodegenerative diseases. Curr Opin Pharmacol.

[23] Armstrong MJ, Okun MS (2020). Diagnosis and treatment of Parkinson disease: a review. JAMA.

[24] Yue B, Yang H, Wang J, Ru W, Wu J, Huang Y (2020). Exosome biogenesis, secretion and function of exosomal miRNAs in skeletal muscle myogenesis. Cell Prolif.

[25] Pinto DO, DeMarino C, Pleet ML, Cowen M, Branscome H, Al Sharif S (2019). HTLV-1 extracellular vesicles promote cell-to-cell contact. Front Microbiol.

[26] Liu J, Zhu L, Wang J, Qiu L, Chen Y, Davis RE (2019). Schistosoma japonicum extracellular vesicle miRNA cargo regulates host macrophage functions facilitating parasitism. PLoS Pathog.

[27] de Jong OG, Murphy DE, Mäger I, Willms E, Garcia-Guerra A, Gitz-Francois JJ (2020). A CRISPR-Cas9-based reporter system for single-cell detection of extracellular vesicle-mediated functional transfer of RNA. Nat Commun.

[28] Bordin A, Chirivì M, Pagano F, Milan M, Iuliano M, Scaccia E (2022). Human platelet lysate-derived extracellular vesicles enhance angiogenesis through miR-126. Cell Prolif.

[29] Cerri S, Ghezzi C, Ongari G, Croce S, Avenali M, Zangaglia R (2021). GBA mutations influence the release and pathological effects of small extracellular vesicles from fibroblasts of patients with Parkinson's disease. Int J Mol Sci.

[30] Ragni E, Palombella S, Lopa S, Talò G, Perucca Orfei C, De Luca P (2020). Innovative visualization and quantification of extracellular vesicles interaction with and incorporation in target cells in 3D microenvironments. Cells.

[31] Lino MM, Simões S, Tomatis F, Albino I, Barrera A, Vivien D (2021). Engineered extracellular vesicles as brain therapeutics. J Control Release.

[32] Wilson DM, Cookson MR, Van Den Bosch L, Zetterberg H, Holtzman DM, Dewachter I (2023). Hallmarks of neurodegenerative diseases. Cell.

[33] Sas A, Jones R, Tyor W (2008). Intra-peritoneal injection of polyclonal anti-interferon alpha antibodies cross the blood brain barrier and neutralize interferon alpha. Neurochem Res.

[34] Jager SE, Pallesen LT, Richner M, Harley P, Hore Z, McMahon S (2020). Changes in the transcriptional fingerprint of satellite glial cells following peripheral nerve injury. Glia.

[35] Gao Y, Qin H, Wu D, Liu C, Fang L, Wang J (2021). Walnut peptide WEKPPVSH in alleviating oxidative stress and inflammation in lipopolysaccharide-activated BV-2 microglia via the Nrf2/HO-1 and NF-κB/p38 MAPK pathways. J Biosci Bioeng.

[36] Hossain MM, Toltin AC, Gamba LM, Molina MA (2022). Deltamethrin-evoked ER stress promotes neuroinflammation in the adult mouse hippocampus. Cells.

[37] Hasan R, Humphrey J, Bettencourt C, Newcombe J, Consortium NA, Lashley T (2021). Transcriptomic analysis of frontotemporal lobar degeneration with TDP-43 pathology reveals cellular alterations across multiple brain regions. Acta Neuropathol.

[38] Marinelli S, Basilico B, Marrone MC, Ragozzino D (2019). Microglia-neuron crosstalk: Signaling mechanism and control of synaptic transmission. Seminars in cell & developmental biology: Elsevier.

[39] Wang J, Hou Y, Zhang L, Liu M, Zhao J, Zhang Z (2021). Estrogen attenuates traumatic brain injury by inhibiting the activation of microglia and astrocyte-mediated neuroinflammatory responses. Mol Neurobiol.

[40] Wang J, Cheng C, Liu Z, Lin Y, Yang L, Zhang Z (2022). Inhibition of A1 Astrocytes and Activation of A2 Astrocytes for the Treatment of Spinal Cord Injury. Neurochem Res.

[41] Han J, Chitu V, Stanley ER, Wszolek ZK, Karrenbauer VD, Harris RA (2022). Inhibition of colony stimulating factor-1 receptor (CSF-1R) as a potential therapeutic strategy for neurodegenerative diseases: opportunities and challenges. Cell Mol Life Sci.

[42] Lubetzki C, Sol-Foulon N, Desmazières A (2020). Nodes of Ranvier during development and repair in the CNS. Nat Rev Neurol.

[43] Lv B, Zhang X, Yuan J, Chen Y, Ding H, Cao X (2021). Biomaterial-supported MSC transplantation enhances cell-cell communication for spinal cord injury. Stem Cell Res Ther.

[44] Mecha M, Yanguas-Casás N, Feliú A, Mestre L, Carrillo-Salinas FJ, Riecken K (2020). Involvement of Wnt7a in the role of M2c microglia in neural stem cell oligodendrogenesis. J Neuroinflammation.

[45] Traiffort E, Kassoussi A, Zahaf A, Laouarem Y (2020). Astrocytes and microglia as major players of myelin production in normal and pathological conditions. Front Cell Neurosci.

[46] Correale J, Marrodan M, Ysrraelit MC (2019). Mechanisms of neurodegeneration and axonal dysfunction in progressive multiple sclerosis. Biomedicines.

[47] Xu J, Zhao J, Wang R, Zhang Y, Shen L, Xiao Q (2022). Shh and Olig2 sequentially regulate oligodendrocyte differentiation from hiPSCs for the treatment of ischemic stroke. Theranostics.

[48] Ye L-X, An N-C, Huang P, Li D-H, Zheng Z-L, Ji H (2021). Exogenous platelet-derived growth factor improves neurovascular unit recovery after spinal cord injury. Neural Regen Res.

[49] Huang H-T, Ho C-H, Sung H-Y, Lee L-Y, Chen W-P, Chen Y-W (2021). Hericium erinaceus mycelium and its small bioactive compounds promote oligodendrocyte maturation with an increase in myelin basic protein. Sci Rep.

[50] Baror R, Neumann B, Segel M, Chalut KJ, Fancy SP, Schafer DP (2019). Transforming growth factor-beta renders ageing microglia inhibitory to oligodendrocyte generation by CNS progenitors. Glia.

[51] El Waly B, Buttigieg E, Karakus C, Brustlein S, Debarbieux F (2020). Longitudinal intravital microscopy reveals axon degeneration concomitant with inflammatory cell infiltration in an LPC model of demyelination. Front Cell Neurosci.

[52] Xiao Y, Zhang Y, Gao Y-H, Zhao Z-H, He J, Gao R (2022). A targeted extracellular vesicles loaded with montelukast in the treatment of demyelinating diseases. Biochem Biophys Res Commun.

[53] Drijgers RL, Aalten P, Winogrodzka A, Verhey FR, Leentjens AF (2009). Pharmacological treatment of apathy in neurodegenerative diseases: a systematic review. Dement Geriatr Cogn Disord.

[54] Arbesman M, Lieberman D, Berlanstein DR (2014). Method for the systematic reviews on occupational therapy and neurodegenerative diseases. Am J Occup Ther.

[55] Tye CB, Gardner PA, Dion GR, Simpson CB, Dominguez LM (2021). Impact of fiberoptic endoscopic evaluation of swallowing outcomes and dysphagia management in neurodegenerative diseases. Laryngoscope.

[56] Armitage A, Fonkem E (2023). Supportive care of neurodegenerative patients. Front Oncol.

[57] Chandrasekaran S, Veeran R (2020). Assistive Device for Neurodegenerative Disease Patients Using IoT. Innovative Data Communication Technologies and Application: ICIDCA 2019: Springer.

[58] Santiago JA, Potashkin JA (2023). Physical activity and lifestyle modifications in the treatment of neurodegenerative diseases. Front Aging Neurosci.

[59] Lee S, Bang SM, Lee JW, Cho KS (2014). Evaluation of traditional medicines for neurodegenerative diseases using Drosophila models. Evid Based Complementary Altern Med. 2014.

[60] Ramanathan S, Archunan G, Sivakumar M, Selvan ST, Fred AL, Kumar S (2018). Theranostic applications of nanoparticles in neurodegenerative disorders. Int J Nanomedicine.

[61] Vissers MF, Heuberger JA, Groeneveld GJ (2021). Targeting for success: demonstrating proof-of-concept with mechanistic early phase clinical pharmacology studies for disease-modification in neurodegenerative disorders. Int J Mol Sci.

[62] Speed LJ, van Dam WO, Hirath P, Vigliocco G, Desai RH (2017). Impaired comprehension of speed verbs in Parkinson's disease. J Int Neuropsychol Soc.

[63] Modi G, Pillay V, Choonara YE (2010). Advances in the treatment of neurodegenerative disorders employing nanotechnology. Ann N Y Acad Sci.

[64] Naziris N, Demetzos C (2021). Advanced Health Technologies and Nanotechnologies in Neurodegenerative Diseases. GeNeDis 2020: Genetics and Neurodegenerative Diseases: Springer.

[65] Sudhakar V, Richardson RM (2019). Gene therapy for neurodegenerative diseases. Neurotherapeutics.

[66] D'Souza GX, Rose SE, Knupp A, Nicholson DA, Keene CD, Young JE (2021). The application of *in vitro*-derived human neurons in neurodegenerative disease modeling. J Neurosci Res.

[67] Piguet F, de Saint Denis T, Audouard E, Beccaria K, André A, Wurtz G (2021). The challenge of gene therapy for neurological diseases: strategies and tools to achieve efficient delivery to the central nervous system. Hum Gene Ther.

[68] Richardson RM, Larson P (2019). Direct Convective Nervous System Drug Delivery for Patients with Neurodegenerative Disorders. Nervous System Drug Delivery: Elsevier.

[69] Rosser AE, Busse ME, Gray WP, Badin RA, Perrier AL, Wheelock V (2022). Translating cell therapies for neurodegenerative diseases: Huntington's disease as a model disorder. Brain.

[70] Bonfili L, Cecarini V, Gogoi O, Gong C, Cuccioloni M, Angeletti M (2021). Microbiota modulation as preventative and therapeutic approach in Alzheimer's disease. FEBS J.

[71] Akwa Y (2020). Steroids and Alzheimer's disease: changes associated with pathology and therapeutic potential. Int J Mol Sci.

[72] Ouanes S, Clark C, Richiardi J, Maréchal B, Lewczuk P, Kornhuber J (2022). Cerebrospinal fluid cortisol and dehydroepiandrosterone sulfate, Alzheimer's disease pathology, and cognitive decline. Front Aging Neurosci.

[73] Deeb O, Nabulsi M (2020). Exploring Multiple Sclerosis (MS) and Amyotrophic Lateral Scler osis (ALS) as neurodegenerative diseases and their treatments: a review study. Curr Top Med Chem.

[74] Renaud J, Martinoli M-G (2019). Considerations for the use of polyphenols as therapies in neurodegenerative diseases. Int J Mol Sci.

[75] Mortada I, Farah R, Nabha S, Ojcius DM, Fares Y, Almawi WY (2021). Immunotherapies for neurodegenerative diseases. Front Neurol.

[76] Shin J, Kim H-J, Jeon B (2020). Immunotherapy targeting neurodegenerative proteinopathies: α-synucleinopathies and tauopathies. J Mov Disord.

[77] Kwon S, Iba M, Kim C, Masliah E (2020). Immunotherapies for aging-related neurodegenerative diseases—emerging perspectives and new targets. Neurotherapeutics.

[78] Wang S, Colonna M (2019). Microglia in Alzheimer's disease: a target for immunotherapy. J Leukoc Biol.

[79] Manjula R, Anuja K, Alcain FJ (2021). SIRT1 and SIRT2 activity control in neurodegenerative diseases. Front Pharmacol.

[80] Gorabi AM, Kiaie N, Barreto GE, Read MI, Tafti HA, Sahebkar A (2019). The therapeutic potential of mesenchymal stem cell-derived exosomes in treatment of neurodegenerative diseases. Mol Neurobiol.

[81] Krauss R, Bosanac T, Devraj R, Engber T, Hughes RO (2020). Axons matter: the promise of treating neurodegenerative disorders by targeting SARM1-mediated axonal degeneration. Trends Pharmacol Sci.

[82] Vijiaratnam N, Foltynie T (2020). Therapeutic strategies to treat or prevent off episodes in adults with Parkinson's disease. Drugs.

[83] Mullane K, Williams M (2020). Alzheimer's disease beyond amyloid: Can the repetitive failures of amyloid-targeted therapeutics inform future approaches to dementia drug discovery?. Biochem Pharmacol.

[84] Liu D, Zhang Q, Luo P, Gu L, Shen S, Tang H (2022). Neuroprotective Effects of Celastrol in Neurodegenerative Diseases-Unscramble Its Major Mechanisms of Action and Targets. Aging Dis.

[85] Krzysztoforska K, Mirowska-Guzel D, Widy-Tyszkiewicz E (2019). Pharmacological effects of protocatechuic acid and its therapeutic potential in neurodegenerative diseases: Review on the basis of *in vitro* and *in vivo* studies in rodents and humans. Nutr Neurosci.

[86] Abdel-Salam OM (2008). Drugs used to treat Parkinson's disease, present status and future directions. CNS Neurol Disord Drug Targets.

[87] Scatena R, Martorana GE, Bottoni P, Botta G, Pastore P, Giardina B (2007). An update on pharmacological approaches to neurodegenerative diseases. Expert Opin Investig Drugs.

[88] Marucci G, Buccioni M, Dal Ben D, Lambertucci C, Volpini R, Amenta F (2021). Efficacy of acetylcholinesterase inhibitors in Alzheimer's disease. Neuropharmacology.

[89] Molino I, Colucci L, Fasanaro AM, Traini E, Amenta F (2013). Efficacy of memantine, donepezil, or their association in moderate-severe Alzheimer's disease: a review of clinical trials. Sci World J. 2013.

[90] Verma A, Waiker DK, Bhardwaj B, Saraf P, Shrivastava SK The molecular mechanism, targets, and novel molecules in the treatment of Alzheimer's disease. Bioorg Chem. 2021: 105562.

[91] Cummings J (2021). New approaches to symptomatic treatments for Alzheimer's disease. Mol Neurodegener.

[92] Patyar S, Prakash A, Medhi B (2011). Dual inhibition: a novel promising pharmacological approach for different disease conditions. J Pharm Pharmacol.

[93] Zec RF, Burkett NR (2008). Non-pharmacological and pharmacological treatment of the cognitive and behavioral symptoms of Alzheimer disease. NeuroRehabilitation.

[94] Moreira NCdS, Lima JEBdF, Marchiori MF, Carvalho I, Sakamoto-Hojo ET (2022). Neuroprotective effects of cholinesterase inhibitors: current scenario in therapies for alzheimer's disease and future perspectives. J Alzheimers Dis Rep.

[95] Tanna T, Sachan V (2014). Mesenchymal stem cells: potential in treatment of neurodegenerative diseases. Curr Stem Cell Res Ther.

[96] Yao P, Zhou L, Zhu L, Zhou B, Yu Q (2020). Mesenchymal stem cells: a potential therapeutic strategy for neurodegenerative diseases. Eur Neurol.

[97] Zakrzewski W, Dobrzyński M, Szymonowicz M, Rybak Z (2019). Stem cells: past, present, and future. Stem Cell Res Ther.

[98] Scuteri A, Miloso M, Foudah D, Orciani M, Cavaletti G, Tredici G (2011). Mesenchymal stem cells neuronal differentiation ability: a real perspective for nervous system repair?. Curr Stem Cell Res Ther.

[99] Hendijani F (2017). Explant culture: An advantageous method for isolation of mesenchymal stem cells from human tissues. Cell Prolif.

[100] Nawab K, Bhere D, Bommarito A, Mufti M, Naeem A (2019). Stem cell therapies: a way to promising cures. Cureus.

[101] Debosschere Y, Depuydt E, Pauwelyn G, Beerts C, Van Hecke L, Verhaert L (2020). Safety and immunomodulatory properties of equine peripheral blood-derived mesenchymal stem cells in healthy cats. Vet Immunol Immunopathol.

[102] Kumar P, Kandoi S, Misra R, Vijayalakshmi S, Rajagopal K, Verma RS (2019). The mesenchymal stem cell secretome: A new paradigm towards cell-free therapeutic mode in regenerative medicine. Cytokine Growth Factor Rev.

[103] Zomer HD, da Silva Jeremias T, Ratner B, Trentin AG (2020). Mesenchymal stromal cells from dermal and adipose tissues induce macrophage polarization to a pro-repair phenotype and improve skin wound healing. Cytotherapy.

[104] Mohamed SA, Duffy A, McInerney V, Krawczyk J, Hayat A, Naughton S (2022). Marrow changes and reduced proliferative capacity of mesenchymal stromal cells from patients with “no-option” critical limb ischemia; observations on feasibility of the autologous approach from a clinical trial. Cytotherapy.

[105] Vatsa P, Negi R, Ansari U, Khanna V, Pant A (2022). Insights of extracellular vesicles of mesenchymal stem cells: a prospective cell-free regenerative medicine for neurodegenerative disorders. Mol Neurobiol.

[106] Monakova A, Sagaradze G, Basalova N, Popov V, Balabanyan V, Efimenko A (2022). Novel Potency Assay for MSC Secretome-Based Treatment of Idiopathic Male Infertility Employed Leydig Cells and Revealed Vascular Endothelial Growth Factor as a Promising Potency Marker. IntJ MolSci.

[107] Yu H, Cheng J, Shi W, Ren B, Zhao F, Shi Y (2020). Bone marrow mesenchymal stem cell-derived exosomes promote tendon regeneration by facilitating the proliferation and migration of endogenous tendon stem/progenitor cells. Acta Biomater.

[108] Alfaro M, Majcherczyk A, Kües U, Ramírez L, Pisabarro AG (2020). Glucose counteracts wood-dependent induction of lignocellulolytic enzyme secretion in monokaryon and dikaryon submerged cultures of the white-rot basidiomycete Pleurotus ostreatus. Sci Rep.

[109] Geetha N, Sunilkumar CR, Bhavya G, Nandini B, Abhijith P, Satapute P Warhorses in soil bioremediation: Seed biopriming with PGPF secretome to phytostimulate crop health under heavy metal stress. Environ Res. 2022: 114498.

[110] Chen X, Lu W, Zuo Y, Ye J, Li X, Wu Z (2022). Injectable hydrogel embedded with mesenchymal stem cells repairs severe spinal cord injury. bioRxiv. 2022.

[111] Yousefi F, Arab FL, Saeidi K, Amiri H, Mahmoudi M (2019). Various strategies to improve efficacy of stem cell transplantation in multiple sclerosis: focus on mesenchymal stem cells and neuroprotection. J Neuroimmunol.

[112] Santamaria G, Brandi E, Vitola PL, Grandi F, Ferrara G, Pischiutta F (2021). Intranasal delivery of mesenchymal stem cell secretome repairs the brain of Alzheimer's mice. Cell Death Diffe.

[113] Phillips W, Willms E, Hill AF (2021). Understanding extracellular vesicle and nanoparticle heterogeneity: Novel methods and considerations. Proteomics.

[114] Morgan TK (2018). Cell-and size-specific analysis of placental extracellular vesicles in maternal plasma and pre-eclampsia. Transl Res.

[115] Shao H, Im H, Castro CM, Breakefield X, Weissleder R, Lee H (2018). New technologies for analysis of extracellular vesicles. Chem Rev.

[116] Weisgerber AW, Mahmood A, Wehman AM, Knowles MK (2022). Molecular regulation of multivesicular endosome fusion and exosome secretion. Exocytosis: From Molecules to Cells: IOPScience.

[117] Mantile F, Franco P, Stoppelli MP, Liguori GL (2021). Biological role and clinical relevance of extracellular vesicles as key mediators of cell communication in cancer. Advances in Biomembranes and Lipid Self-Assembly: Elsevier.

[119] Buratta S, Tancini B, Sagini K, Delo F, Chiaradia E, Urbanelli L (2020). Lysosomal exocytosis, exosome release and secretory autophagy: the autophagic-and endo-lysosomal systems go extracellular. Int J Mol Sci.

[120] Geng T, Pan P, Leung E, Chen Q, Chamley L, Wu Z (2021). Recent advancement and technical challenges in developing small extracellular vesicles for cancer drug delivery. Pharm Res.

[121] Carberry CK, Keshava D, Payton A, Smith GJ, Rager JE (2022). Approaches to incorporate extracellular vesicles into exposure science, toxicology, and public health research. J Expo Sci Environ Epidemiol.

[122] Mathieu M, Névo N, Jouve M, Valenzuela JI, Maurin M, Verweij FJ (2021). Specificities of exosome versus small ectosome secretion revealed by live intracellular tracking of CD63 and CD9. Nat Commun.

[123] Spencer N, Yeruva L (2021). Role of bacterial infections in extracellular vesicles release and impact on immune response. Biomed J.

[124] Kwok ZH, Wang C, Jin Y (2021). Extracellular vesicle transportation and uptake by recipient cells: A critical process to regulate human diseases. Processes.

[125] Ginini L, Billan S, Fridman E, Gil Z (2022). Insight into extracellular vesicle-cell communication: from cell recognition to intracellular fate. Cells.

[126] van der Koog L, Gandek TB, Nagelkerke A (2022). Liposomes and extracellular vesicles as drug delivery systems: A comparison of composition, pharmacokinetics, and functionalization. Adv Healthc Mater.

[127] Maugeri M, Nawaz M, Papadimitriou A, Angerfors A, Camponeschi A, Na M (2019). Linkage between endosomal escape of LNP-mRNA and loading into EVs for transport to other cells. Nat Commun.

[128] Cronqvist T, Erlandsson L, Tannetta D, Hansson SR (2020). Placental syncytiotrophoblast extracellular vesicles enter primary endothelial cells through clathrin-mediated endocytosis. Placenta.

[129] Rai AK, Johnson PJ (2019). Trichomonas vaginalis extracellular vesicles are internalized by host cells using proteoglycans and caveolin-dependent endocytosis. Proc Natl Acad Sci.

[130] Wan S, Sun X, Tang W, Wang L, Wu Z, Sun X (2021). Exosome-depleted excretory-secretory products of the fourth-stage larval angiostrongylus cantonensis promotes alternative activation of macrophages through metabolic reprogramming by the PI3K-Akt pathway. Front Immunol.

[131] Jadli AS, Ballasy N, Edalat P, Patel VB (2020). Inside (sight) of tiny communicator: exosome biogenesis, secretion, and uptake. Mol Cell Biochem.

[132] Varyukhina S, Lamazière A, Delaunay JL, de Wreede A, Ayala-Sanmartin J (2022). The Ca2+-and phospholipid-binding protein Annexin A2 is able to increase and decrease plasma membrane order. Biochim Biophys Acta-Biomembr.

[133] Wei W, Pan Y, Yang X, Chen Z, Heng Y, Yang B (2022). The Emerging Role of the Interaction of Extracellular Vesicle and Autophagy—Novel Insights into Neurological Disorders. J Inflamm Res.

[135] Wang S, Gao Y (2021). Pancreatic cancer cell-derived microRNA-155-5p-containing extracellular vesicles promote immune evasion by triggering EHF-dependent activation of Akt/NF-κB signaling pathway. Int Immunopharmacol.

[136] Veziroglu EM, Mias GI (2020). Characterizing extracellular vesicles and their diverse RNA contents. Front Genet.

[137] Fuhrmann G, Serio A, Mazo M, Nair R, Stevens MM (2015). Active loading into extracellular vesicles significantly improves the cellular uptake and photodynamic effect of porphyrins. J Control Release.

[138] Raffaele S, Lombardi M, Verderio C, Fumagalli M (2020). TNF production and release from microglia via extracellular vesicles: impact on brain functions. Cells.

[139] Koniusz S, Andrzejewska A, Muraca M, Srivastava AK, Janowski M, Lukomska B (2016). Extracellular vesicles in physiology, pathology, and therapy of the immune and central nervous system, with focus on extracellular vesicles derived from mesenchymal stem cells as therapeutic tools. Front Cell Neurosci.

[140] Liangsupree T, Multia E, Riekkola M-L (2021). Modern isolation and separation techniques for extracellular vesicles. J Chromatogr A.

[141] O'Neil EV, Burns GW, Ferreira CR, Spencer TE (2020). Characterization and regulation of extracellular vesicles in the lumen of the ovine uterus. Biol Reprod.

[142] Wu D, Yan J, Shen X, Sun Y, Thulin M, Cai Y (2019). Profiling surface proteins on individual exosomes using a proximity barcoding assay. Nat Commun.

[143] Sitar S, Kejžar A, Pahovnik D, Kogej K, Tušek-Žnidarič M, Lenassi M (2015). Size characterization and quantification of exosomes by asymmetrical-flow field-flow fractionation. Anal Chem.

[144] Wunsch BH, Smith JT, Gifford SM, Wang C, Brink M, Bruce RL (2016). Nanoscale lateral displacement arrays for the separation of exosomes and colloids down to 20 nm. Nat Nanotechnol.

[145] Deregibus MC, Figliolini F, D'antico S, Manzini PM, Pasquino C, De Lena M (2016). Charge-based precipitation of extracellular vesicles. Int J Mol Med.

[146] Morani M, Mai TD, Krupova Z, Defrenaix P, Multia E, Riekkola M-L (2020). Electrokinetic characterization of extracellular vesicles with capillary electrophoresis: A new tool for their identification and quantification. Anal Chim Acta.

[147] Petersen KE, Shiri F, White T, Bardi GT, Sant H, Gale BK (2018). Exosome isolation: cyclical electrical field flow fractionation in low-ionic-strength fluids. Anal Chem.

[148] Shiri F, Gale BK, Sant H, Bardi GT, Hood JL, Petersen KE (2020). Characterization of human glioblastoma versus normal plasma-derived extracellular vesicles preisolated by differential centrifugation using cyclical electrical field-flow fractionation. Anal Chem.

[149] Théry C, Zitvogel L, Amigorena S (2002). Exosomes: composition, biogenesis and function. Nat Rev Immunol.

[150] Wang L, Bruce TF, Huang S, Marcus RK (2019). Isolation and quantitation of exosomes isolated from human plasma via hydrophobic interaction chromatography using a polyester, capillary-channeled polymer fiber phase. Anal Chim Acta.

[151] Huang S, Wang L, Bruce TF, Marcus RK (2019). Isolation and quantification of human urinary exosomes by hydrophobic interaction chromatography on a polyester capillary-channeled polymer fiber stationary phase. Anal Bioanal Chem.

[152] Ku A, Ravi N, Yang M, Evander M, Laurell T, Lilja H (2019). A urinary extracellular vesicle microRNA biomarker discovery pipeline; from automated extracellular vesicle enrichment by acoustic trapping to microRNA sequencing. PLoS One.

[153] Ku A, Lim HC, Evander M, Lilja H, Laurell T, Scheding S (2018). Acoustic enrichment of extracellular vesicles from biological fluids. Anal Chem.

[154] Nemati Z, Um J, Zamani Kouhpanji MR, Zhou F, Gage T, Shore D (2020). Magnetic isolation of cancer-derived exosomes using Fe/Au magnetic nanowires. ACS Appl Nano Mater.

[155] Colao IL, Corteling R, Bracewell D, Wall I (2018). Manufacturing exosomes: a promising therapeutic platform. Trends Mol Med.

[156] Gandham S, Su X, Wood J, Nocera AL, Alli SC, Milane L (2020). Technologies and standardization in research on extracellular vesicles. Trends Biotechnol.

[157] Lu C-H, Chen Y-A, Ke C-C, Liu R-S (2021). Mesenchymal stem cell-derived extracellular vesicle: a promising alternative therapy for osteoporosis. Int J Mol Sci.

[158] Mendt M, Kamerkar S, Sugimoto H, McAndrews KM, Wu C-C, Gagea M (2018). Generation and testing of clinical-grade exosomes for pancreatic cancer. JCI insight.

[159] Jeyaram A, Jay SM (2018). Preservation and storage stability of extracellular vesicles for therapeutic applications. AAPS J.

[160] Kusuma GD, Barabadi M, Tan JL, Morton DA, Frith JE, Lim R (2018). To protect and to preserve: novel preservation strategies for extracellular vesicles. Front Pharmacol.

[161] Charoenviriyakul C, Takahashi Y, Nishikawa M, Takakura Y (2018). Preservation of exosomes at room temperature using lyophilization. Int J Pharm.

[162] Laggner M, Gugerell A, Bachmann C, Hofbauer H, Vorstandlechner V, Seibold M (2020). Reproducibility of GMP-compliant production of therapeutic stressed peripheral blood mononuclear cell-derived secretomes, a novel class of biological medicinal products. Stem Cell Res Ther.

[163] Richards A, Krakowka S, Dexter L, Schmid H, Wolterbeek A, Waalkens-Berendsen D (2002). Trehalose: a review of properties, history of use and human tolerance, and results of multiple safety studies. Food Chem. Toxicol.

[164] Romanov YA, Volgina N, Dugina T, Kabaeva N, Sukhikh G (2019). Effect of storage conditions on the integrity of human umbilical cord mesenchymal stromal cell-derived microvesicles. Bull Exp Biol Med.

[165] Lőrincz ÁM, Timar CI, Marosvari KA, Veres DS, Otrokocsi L, Kittel A (2014). Effect of storage on physical and functional properties of extracellular vesicles derived from neutrophilic granulocytes. J Extracell Vesicles.

[166] Liu S, Gao Y, Zhou D, Zeng M, Alshehri F, Newland B (2019). Highly branched poly (β-amino ester) delivery of minicircle DNA for transfection of neurodegenerative disease related cells. Nat Commun.

[167] Farahzadi R, Fathi E, Vietor I (2020). Mesenchymal stem cells could be considered as a candidate for further studies in cell-based therapy of Alzheimer's disease via targeting the signaling pathways. ACS Chem Neurosci.

[168] Yan Y-C, Li Y-H, Xiao B-G, Xi J-Y, Yu W-B Synergetic Therapeutic Efficacy of Fasudil and Bone Marrow Derived-Neuronal Stem Cell in a Parkinson's Disease Mouse Model. 2022.

[169] Qiu L, Cai Y, Geng Y, Yao X, Wang L, Cao H (2022). Mesenchymal stem cell-derived extracellular vesicles attenuate tPA-induced blood-brain barrier disruption in murine ischemic stroke models. Acta Biomater.

[170] Liu H, Liang J, Ye X, Huang M, Ma L, Xie X (2022). The potential role of extracellular vesicles in bioactive compound-based therapy: A review of recent developments. Crit Rev Food Sci Nutr.

[171] Malina T, Poláková K, Skopalík J, Milotová V, Holá K, Havrdová M (2019). Carbon dots for *in vivo* fluorescence imaging of adipose tissue-derived mesenchymal stromal cells. Carbon.

[172] Yamada Y, Nakamura-Yamada S, Kusano K, Baba S (2019). Clinical potential and current progress of dental pulp stem cells for various systemic diseases in regenerative medicine: a concise review. Int J Mol Sci.

[173] Carter K, Lee HJ, Na K-S, Fernandes-Cunha GM, Blanco IJ, Djalilian A (2019). Characterizing the impact of 2D and 3D culture conditions on the therapeutic effects of human mesenchymal stem cell secretome on corneal wound healing *in vitro* and *ex vivo*. Acta Biomater.

[174] Soares Martins T, Marçalo R, Ferreira M, Vaz M, Silva RM, Martins Rosa I (2021). Exosomal Aβ-binding proteins identified by “*In Silico*” analysis represent putative blood-derived biomarker candidates for Alzheimer´ s disease. Int J Mol Sci.

[175] Bang OY, Kim J-E (2022). Stem cell-derived extracellular vesicle therapy for acute brain insults and neurodegenerative diseases. BMB Rep.

[176] Sahraian MA, Mohyeddin Bonab M, Baghbanian SM, Owji M, Naser Moghadasi A (2019). Therapeutic use of intrathecal mesenchymal stem cells in patients with multiple sclerosis: a pilot study with booster injection. Immunol Invest.

[177] Des Rieux A (2021). Stem cells and their extracellular vesicles as natural and bioinspired carriers for the treatment of neurological disorders. Curr Opin Colloid Interface Sci.

[178] Raffo-Romero A, Arab T, Al-Amri IS, Le Marrec-Croq F, Van Camp C, Lemaire Q (2018). Medicinal leech CNS as a model for exosome studies in the crosstalk between microglia and neurons. Int J Mol Sci.

[179] Mallawaaratchy DM, Hallal S, Russell B, Ly L, Ebrahimkhani S, Wei H (2017). Comprehensive proteome profiling of glioblastoma-derived extracellular vesicles identifies markers for more aggressive disease. J Neurooncol.

[180] Sharma K, Zhang Y, Paudel K, Kachelmeier A, Hansbro P, Shi X (2022). The Emerging Role of Pericyte-Derived Extracellular Vesicles in Vascular and Neurological Health. Cells.

[181] Shi X, Zhong X, Deng L, Wu X, Zhang P, Zhang X (2022). Mesenchymal Stem Cell-derived Extracellular Vesicle-enclosed microRNA-93 Prevents Hypoxic-ischemic Brain Damage in Rats. Neuroscience.

[182] Wang S-S, Jia J, Wang Z (2018). Mesenchymal stem cell-derived extracellular vesicles suppresses iNOS expression and ameliorates neural impairment in Alzheimer's disease mice. J Alzheimer's Dis.

[183] Sha S, Shen X, Cao Y, Qu L (2021). Mesenchymal stem cells-derived extracellular vesicles ameliorate Alzheimer's disease in rat models via the microRNA-29c-3p/BACE1 axis and the Wnt/β-catenin pathway. Aging.

[184] Elia CA, Tamborini M, Rasile M, Desiato G, Marchetti S, Swuec P (2019). Intracerebral injection of extracellular vesicles from mesenchymal stem cells exerts reduced Aβ plaque burden in early stages of a preclinical model of Alzheimer's disease. Cells.

[185] Ye X, Sun X, Starovoytov V, Cai Q (2015). Parkin-mediated mitophagy in mutant hAPP neurons and Alzheimer's disease patient brains. Hum Mol Genet.

[186] Xu F, Wu Y, Yang Q, Cheng Y, Xu J, Zhang Y Engineered Extracellular Vesicles with SHP2 High Expression Promote Mitophagy for Alzheimer's Disease Treatment. Adv Mater. 2022: 2207107.

[187] Losurdo M, Pedrazzoli M, D'Agostino C, Elia CA, Massenzio F, Lonati E (2020). Intranasal delivery of mesenchymal stem cell-derived extracellular vesicles exerts immunomodulatory and neuroprotective effects in a 3xTg model of Alzheimer's disease. Stem Cells Transl Med.

[188] Kaniowska D, Wenk K, Rademacher P, Weiss R, Fabian C, Schulz I (2022). Extracellular vesicles of mesenchymal stromal cells can be taken up by microglial cells and partially prevent the stimulation induced by β-amyloid. Stem Cell Rev Rep.

[189] Zhang Z, Sheng H, Liao L, Xu C, Zhang A, Yang Y (2020). Mesenchymal stem cell-conditioned medium improves mitochondrial dysfunction and suppresses apoptosis in okadaic acid-treated SH-SY5Y cells by extracellular vesicle mitochondrial transfer. J Alzheimer's Dis.

[190] Provenzano F, Nyberg S, Giunti D, Torazza C, Parodi B, Bonifacino T (2022). Micro-RNAs Shuttled by Extracellular Vesicles Secreted from Mesenchymal Stem Cells Dampen Astrocyte Pathological Activation and Support Neuroprotection in In-Vitro Models of ALS. Cells.

[191] Markoutsa E, Mayilsamy K, Gulick D, Mohapatra SS, Mohapatra S (2022). Extracellular vesicles derived from inflammatory-educated stem cells reverse brain inflammation—implication of miRNAs. Mol Ther.

[192] Long Q, Upadhya D, Hattiangady B, Kim D-K, An SY, Shuai B (2017). Intranasal MSC-derived A1-exosomes ease inflammation, and prevent abnormal neurogenesis and memory dysfunction after status epilepticus. Proc Natl Acad Sci.

[193] Laso-García F, Ramos-Cejudo J, Carrillo-Salinas FJ, Otero-Ortega L, Feliú A, Gómez-de Frutos M (2018). Therapeutic potential of extracellular vesicles derived from human mesenchymal stem cells in a model of progressive multiple sclerosis. PloS one.

[194] Giunti D, Marini C, Parodi B, Usai C, Milanese M, Bonanno G (2021). Role of miRNAs shuttled by mesenchymal stem cell-derived small extracellular vesicles in modulating neuroinflammation. Sci Rep.

[195] Clark K, Zhang S, Barthe S, Kumar P, Pivetti C, Kreutzberg N (2019). Placental mesenchymal stem cell-derived extracellular vesicles promote myelin regeneration in an animal model of multiple sclerosis. Cells.

[196] Kaminski N, Köster C, Mouloud Y, Börger V, Felderhoff-Müser U, Bendix I Mesenchymal stromal cell-derived extracellular vesicles reduce neuroinflammation, promote neural cell proliferation and improve oligodendrocyte maturation in neonatal hypoxic-ischemic brain injury. Front Cell Neurosci. 2020: 438.

[197] Cone AS, Yuan X, Sun L, Duke LC, Vreones MP, Carrier AN (2021). Mesenchymal stem cell-derived extracellular vesicles ameliorate Alzheimer's disease-like phenotypes in a preclinical mouse model. Theranostics.

[198] Matheakakis A, Batsali A, Papadaki HA, Pontikoglou CG (2021). Therapeutic implications of mesenchymal stromal cells and their extracellular vesicles in autoimmune diseases: from biology to clinical applications. Int J Mol Sci.

[199] Noh MY, Lim SM, Oh K-W, Cho K-A, Park J, Kim K-S (2016). Mesenchymal stem cells modulate the functional properties of microglia via TGF-β secretion. Stem Cells Transl Med.

[200] Atlante A, Valenti D, Latina V, Amadoro G (2022). Dysfunction of Mitochondria in Alzheimer's Disease: ANT and VDAC Interact with Toxic Proteins and Aid to Determine the Fate of Brain Cells. Int J Mol Sci.

[201] Matteo V, Esposito E (2003). Biochemical and therapeutic effects of antioxidants in the treatment of Alzheimer's disease, Parkinson's disease, and amyotrophic lateral sclerosis. Curr Drug Targets CNS Neurol Disord.

[202] Kim HG, Moon M, Choi JG, Park G, Kim A-J, Hur J (2014). Donepezil inhibits the amyloid-beta oligomer-induced microglial activation *in vitro* and *in vivo*. Neurotoxicology.

[203] Stonesifer C, Corey S, Ghanekar S, Diamandis Z, Acosta SA, Borlongan CV (2017). Stem cell therapy for abrogating stroke-induced neuroinflammation and relevant secondary cell death mechanisms. Prog Neurobiol.

[204] Chen S-Y, Lin M-C, Tsai J-S, He P-L, Luo W-T, Herschman H (2019). EP4 antagonist-elicited extracellular vesicles from mesenchymal stem cells rescue cognition/learning deficiencies by restoring brain cellular functions. Stem Cells Transl Med.

[205] Mushahary D, Spittler A, Kasper C, Weber V, Charwat V (2018). Isolation, cultivation, and characterization of human mesenchymal stem cells. Cytom Part A.

[206] Terunuma A, Yoshioka Y, Sekine T, Takane T, Shimizu Y, Narita S (2021). Extracellular vesicles from mesenchymal stem cells of dental pulp and adipose tissue display distinct transcriptomic characteristics suggestive of potential therapeutic targets. J Stem Cells Regen Med.

[207] Chen Q, Xi X, Zeng Y, He Z, Zhao J, Li Y (2019). Acteoside inhibits autophagic apoptosis of retinal ganglion cells to rescue glaucoma-induced optic atrophy. J Cell Biochem.

[208] Mead B, Tomarev S (2017). Bone marrow-derived mesenchymal stem cells-derived exosomes promote survival of retinal ganglion cells through miRNA-dependent mechanisms. Stem Cells Transl Med.

[209] Herz J, Köster C, Reinboth BS, Dzietko M, Hansen W, Sabir H (2018). Interaction between hypothermia and delayed mesenchymal stem cell therapy in neonatal hypoxic-ischemic brain injury. Brain Behav Immun.

[210] Maddineni P, Kasetti RB, Patel PD, Millar JC, Kiehlbauch C, Clark AF (2020). CNS axonal degeneration and transport deficits at the optic nerve head precede structural and functional loss of retinal ganglion cells in a mouse model of glaucoma. Mol Neurodegener.

[211] Seyedrazizadeh S-Z, Poosti S, Nazari A, Alikhani M, Shekari F, Pakdel F (2020). Extracellular vesicles derived from human ES-MSCs protect retinal ganglion cells and preserve retinal function in a rodent model of optic nerve injury. Stem Cell Res Ther.

[212] Cao J, Wang B, Tang T, Lv L, Ding Z, Li Z (2020). Three-dimensional culture of MSCs produces exosomes with improved yield and enhanced therapeutic efficacy for cisplatin-induced acute kidney injury. Stem Cell Res Ther.

[213] Herman S, Fishel I, Offen D (2021). Intranasal delivery of mesenchymal stem cells-derived extracellular vesicles for the treatment of neurological diseases. Stem Cells.

[214] Kodali M, Castro OW, Kim D-K, Thomas A, Shuai B, Attaluri S (2019). Intranasally administered human MSC-derived extracellular vesicles pervasively incorporate into neurons and microglia in both intact and status epilepticus injured forebrain. Int J Mol Sci.

[215] Yuan Y, Sun J, You T, Shen W, Xu W, Dong Q (2022). Extracellular Vesicle-Based Therapeutics in Neurological Disorders. Pharmaceutics.

[216] Sharma P, Mesci P, Carromeu C, McClatchy DR, Schiapparelli L, Yates III JR (2019). Exosomes regulate neurogenesis and circuit assembly. Proc Natl Acad Sci.

[217] El Andaloussi S, Mäger I, Breakefield XO, Wood MJ (2013). Extracellular vesicles: biology and emerging therapeutic opportunities. Nat Rev Drug Discov.

[218] Mendt M, Rezvani K, Shpall E (2019). Mesenchymal stem cell-derived exosomes for clinical use. Bone marrow transplant.

[219] Johnson J, Shojaee M, Mitchell Crow J, Khanabdali R (1963). From mesenchymal stromal cells to engineered extracellular vesicles: a new therapeutic paradigm. Front Cell Dev Biol. 2021.

[220] Zhou T, Yuan Z, Weng J, Pei D, Du X, He C (2021). Challenges and advances in clinical applications of mesenchymal stromal cells. J Hematol Oncol.

[221] de Almeida Fuzeta M, Bernardes N, Oliveira FD, Costa AC, Fernandes-Platzgummer A, Farinha JP (2020). Scalable production of human mesenchymal stromal cell-derived extracellular vesicles under serum-/xeno-free conditions in a microcarrier-based bioreactor culture system. Front Cell Dev Biol.

[222] Khan H, Pan J-J, Li Y, Zhang Z, Yang G-Y (2021). Native and bioengineered exosomes for ischemic stroke therapy. Front Cell Dev Biol.

[223] Banks WA, Sharma P, Bullock KM, Hansen KM, Ludwig N, Whiteside TL (2020). Transport of extracellular vesicles across the blood-brain barrier: brain pharmacokinetics and effects of inflammation. Int J Mol Sci.

[224] Nooshabadi VT, Mardpour S, Yousefi-Ahmadipour A, Allahverdi A, Izadpanah M, Daneshimehr F (2018). The extracellular vesicles-derived from mesenchymal stromal cells: A new therapeutic option in regenerative medicine. J Cell Biochem.

[225] Lin Y, Lu Y, Li X (2020). Biological characteristics of exosomes and genetically engineered exosomes for the targeted delivery of therapeutic agents. J Drug Target.

[226] Jiang Y, Li J, Xue X, Yin Z, Xu K, Su J (2022). Engineered extracellular vesicles for bone therapy. Nano Today.

[227] Rankin-Turner S, Vader P, O'Driscoll L, Giebel B, Heaney LM, Davies OG (2021). A call for the standardised reporting of factors affecting the exogenous loading of extracellular vesicles with therapeutic cargos. Adv Drug Del Rev.

[228] Long Q, Zheng P, Zheng X, Li W, Hua L, Yang Z (2022). Engineered bacterial membrane vesicles are promising carriers for vaccine design and tumor immunotherapy. Adv Drug Del Rev.

[229] Wang J, Ma P, Kim DH, Liu B-F, Demirci U (2021). Towards microfluidic-based exosome isolation and detection for tumor therapy. Nano Today.

[230] Lazar SV, Mor S, Wang D, Goldbloom-Helzner L, Clark K, Hao D (2022). Engineering extracellular vesicles for Alzheimer's disease: An emerging cell-free approach for earlier diagnosis and treatment. WIREs Mech Dis.

[231] Bilousova T, Simmons BJ, Knapp RR, Elias CJ, Campagna J, Melnik M (2020). Dual neutral sphingomyelinase-2/acetylcholinesterase inhibitors for the treatment of Alzheimer's disease. ACS Chem Biol.

